# Development of LMS and Z Score Growth References for Egyptian Children From Birth Up to 5 Years

**DOI:** 10.3389/fped.2020.598499

**Published:** 2021-01-18

**Authors:** Ali M. El Shafie, Fady M. El-Gendy, Dalia M. Allahony, Hossam H. Hegran, Zein A. Omar, Mohamed A. Samir, Zeinab A. Kasemy, Ahmed N. El-Bazzar, Mohamed A. Abd El-Fattah, Amir A. Abdel Monsef, Amir M. Kairallah, Hythem M. Raafet, Ghada M. Baza, Amany G. Salah, Walaa S. Galab, Safa H. Alkalash, Amal A. Salama, Nagwa A. Farag, Wael A. Bahbah

**Affiliations:** ^1^Department of Pediatrics, Faculty of Medicine, Menoufia University, Shebin El-Kom, Egypt; ^2^Department of Public Health and Community Medicine, Faculty of Medicine, Menoufia University, Shebin El-Kom, Egypt; ^3^Ministry of Health Hospitals, Cairo, Egypt; ^4^Family Medicine Department, Faculty of Medicine, Menoufia University, Shebin El-Kom, Egypt

**Keywords:** Egyptian, growth parameters, nutritional status, z score, preschool children

## Abstract

**Background:** The Lambda-Mu-Sigma (LMS) and Z score methods are important for assessment of growth and nutritional status. In Egypt, there is a lack of this tool for monitoring growth in preschool children.

**Objective:** To develop LMS and Z score growth references for assessment of growth and nutritional status for Egyptian children from birth up to 5 years.

**Methods:** A total of 27,537 children [13,888 boys (50.4%) and 13,649 girls (49.6%)] from birth up to 5 years were included in a multistage cross sectional randomized study from different Egyptian geographic districts to create LMS and Z score references for weight, length/height, and body mass index corresponding to age in addition to weight for length/height. Healthy term infants and children, exclusive breast feeding for at least 4 months and not suffering from any chronic diseases were included in this study. Children with dysmorphic features, preterm infants, admitted in neonatal or pediatric intensive care units and having any chronic diseases (hematological, cardiac, hepatic, and renal) were excluded. In addition any health condition that affects child growth including nutritional disorders was also excluded. Un-paired *t*-test was calculated to compare the means of weight for age, length/height for age, weight for length/height, and BMI for-age z scores of the Egyptian and WHO reference values.

**Results:** Through detailed tables and graphs, LMS and Z scores for weight for age, length/height for age, weight for length/height, and BMI for age of both sexes were represented. Our findings showed no statistically significant difference between reference charts of WHO and Egyptian Z score charts (*P* > 0.05).

**Conclusion:** This study provides the first reference for Egyptian children from birth up to 5 years based on Z score tool for assessment the growth and nutritional status in various clinical conditions and research, also allows comparison with references of other countries.

## Introduction

Normal growth is a universal public health concern; growth in pediatric age is the main indicator of health and nutritional status, so it should be done at regular intervals (1). Continuous monitoring of growth may detect any changes before developing permanent damage like behavioral disorders, learning disabilities, and retardation in cognitive developments (2, 3). The designation to diagnose a child with growth impairment needs to be compared to a reference one (4). There are three different systems used to compare a child to the reference; standard deviation scores (Z-scores), percent of median and percentiles. Z-score is widely recognized as the best system for analysis and presentation of anthropometric data owing to its advantages over the other methods and it is the most appropriate descriptor of mal nutrition (5). Growth assessment charts have been developed and matured over 200 years since de Montbeillard's son's growth curve was drawn and this process has involved the interesting interaction between three different disciplines: anthropology for the collection of anthropometry, statistics and graphic design to represent the growth reference as a growth chart (6). Many countries lacking suitable local references for their child growth use international ones (7). The idea of growth standards goes back to recommendations of a Working Group on infant growth established by the WHO, and may be justified for infants who tend to grow similarly under modern conditions (8). Due to genetic, nutritional, health-related, and socioeconomic conditions, there is worldwide variation in growth as well as differences between populations at different ages (9). Khadilkar reported the disadvantage of using global charts such as WHO charts, as they may over diagnose underweight and stunting in a large number of apparently normal children in the developing countries (10).

In 1972, the first national study was conducted on 2,121 children (1,351 males and 770 females) that included a sample of Egyptian children from one governorate (Cairo city) aged 6–18 years. Weight, stature, and body mass index (BMI) were measured and only the percentile methods was used for assessment of growth and nutritional status. The limitations of this study are insufficient sample size, which was collected from one governorate, so it does not represent the entire Egypt (11, 12).

The second national study was conducted in 2002, and included 5,245 children aged from birth to 18 years. It used the percentile methods also to assess the growth and nutritional status of Egyptian children, but it wasn't representative of Egypt as all the included children were from Cairo governorate only (13).

In 2020, the largest national study was conducted in Egypt on a 34,822 children aged from 5 to 19 years representing the whole of Egypt and provided references to Lambda-Mu-Sigma (LMS) and Z score for weight, height, and BMI for Egyptian school children and adolescents (14).

Till now; there is no national study conducted in Egypt on ages from 0 to 5 years using Z score method, so the child growth is assessed by WHO growth charts (9). Yet, it is questionable whether WHO growth references could be used for clinical purposes in Egyptian children, particularly as the Egyptian population undergoes major economic transition that may have major effect on infant and child growth. Our study aimed to establish the first Egyptian Z score references to assess growth and nutritional status for Egyptian preschool children from birth up to 5 years including weight, length/height, and BMI corresponding to age, in addition to weight for length/height instead of using references of other countries. Also allows a tool to compare Egyptian children with others.

## Methods and Design

### Participants

From January 2018 to January 2020, a multistage cross sectional randomized study was conducted on 27,537 children from birth up to 5 years. The same methodology used for our publication titled establishment of Z score references of growth parameters for Egyptian school children and adolescents aged 5–19 years was followed (14). All socioeconomic strata were represented with weighted rural-urban representation. A total of 135 randomly selected facilities including nursery, primary care units and centers were visited. The participants and their caregivers were met in the morning in private rooms immediately after receiving the medical advice or vaccination. The most crowded days were chosen to facilitate rapid and massive data collection. The study sample was determined based on Egypt demographic health survey 2015 (15).

The study included healthy term infants and children who were determined by history and clinical examination including infants exclusively breastfed at least 4 months and continued up to 12 months on breastfeeding. Infants and children with dysmorphic features, preterm infants, admitted in neonatal or pediatric intensive care units and having any chronic diseases (hematological, cardiac, hepatic, and renal) were excluded. In addition any health condition that affects child growth including nutritional disorders was also excluded. Mothers were asked about history of breast feeding vs. artificial and any history of medical importance during pregnancy or lactation.

The total number of children eligible to share in the study was 28,048 children. After applying the inclusion and exclusion criteria, 511 were excluded giving a final total sample of 27,537 children who fulfilled the criteria of the study.

### Ethics Approval

Ethical approval (ID: 190118; Ped) was obtained from institutional research board in Menoufia Faculty of Medicine work in accordance with the Declaration of Helsinki. Written consent from each child's parents or guardians for their participation in the study was obtained after explaining the purpose of the study and that is nothing harmful or invasive would be used.

### Measurements and Data Collection

Weight was measured for ages (from 0 to 71 months), recumbent length (for <24 months) and height (from 24 months to 71 months) then BMI was calculated in all ages (from 0 to 71 months). The curves were constructed using all available data (from birth to 71 months) but the final references were truncated at 60 completed months to avoid right-edge effect (16). Identical measuring equipment was used to examine all infants and children. Weight without shoes and heavy outer clothing was measured on a balanced scale (LAICA model bf2051, Italy) till the age of 2 years then another balanced scale (Beurer model GS 11, Germany) was used. The length of children below 2 years of age was measured supine by a platform with an attached fixed head plate, and a movable footplate. The height of those above 2 years was measured upright by Harpenden fixed stadiometer. All devices were calibrated daily. BMI was calculated using the formula: BMI = weight (Kg)/ (height)^2^ (m) (17). The involved field team had been trained over 2 days on all measurements followed by testing to deliver accurate results free of bias.

### Statistical Analysis

L (power in the Box–Cox transformation used to convert the distribution of date to normal distribution), M (Median), and S (the generalized coefficient of variation method) was applied then the standard deviation from −3 to +3 of weight, height/length, BMI for both sexes was determined followed by smoothing of the curve based on method of maximum penalized likelihood. Z score was calculated from the LMS parameters by using the following formula: P = M [1 + LSZ]1/L, L ≠ 0 (18–20).

Z-score was statistically analyzed by using the Statistical package SPSS, version 20, for windows (SPSS Inc., Chicago, Illinois, USA) and Excel. Closeness of the fitted centiles to the observed centiles is the primary criterion for assessing goodness of fit. Comparing the observed and expected centiles of the data Z-scores was performed. Checking of visual impressions were performed by comparing the number of points in each of the regions defined by the centiles with expectation and using Q, test statistic based on the Wilson and Hilferty (21), transformed and recommended by Pan and Cole (22) and described in Hosseini et al. (23). Un-paired *t*-test was calculated to compare the means of weight for age, length/height for age, weight for length/height, and BMI for-age z scores of the Egyptian and WHO reference values. Statistical significance was considered at *P* <0.05.

## Results

A total of 27,537 Egyptian children distributed as 13,888 boys (50.4%) and 13,649 girls (49.6%) with male-female ratio of 1.01. All included children from birth up to 5 years of age were examined in this study for weight for age, length/ height for age, weight for length/height and BMI for age. Moving one standard deviation above and below the mean included 68% of the examined children. 95% are placed within the range of two standard deviations away from the mean. Moving three standard deviations away from the mean included 99.7%. Mean and standard deviation (SD) was for height (87.94 ± 15.24), weight (12.53 ± 3.74), and BMI (15.86 ± 0.83) (Table 1).

**Table 1:** Mean and standard deviation (SD) for weight, Length/Height, and BMI of children aged from birth up to 5 years.

	**n**	**Weight (Kg)**	**Length/height (cm)**	**BMI (kg/m^**2**^)**
		**Mean ± SD**	**Mean ± SD**	**Mean ± SD**
**Boys**				
▪ 1 year (0–12 months)	2,671	7.36, 1.89	62.91, 7.37	17.39, 1.02
▪ 2 year (13–24 months)	2,765	10.74, 0.91	79.11, 3.65	16.41, 0.62
▪ 3 year (25–36 months)	2,931	13.36, 0.68	90.92, 3.67	16.21, 0.38
▪ 4 year (37–48 months)	2,617	15.18, 0.56	97.99, 2.44	15.55, 0.22
▪ 5 year (49–60 months)	2,904	17.021, 0.69	105.17, 2.02	15.23, 0.23
**Girls**				
▪ 1 year (0–12 months)	2,752	6.68, 1.74	64.36, 7.70	16.51, 1.12
▪ 2 year (13–24 months)	2,674	9.80, 0.66	80.86, 3.65	15.67, 0.84
▪ 3 year (25–36 months)	2,925	12.71, 0.79	90.67, 2.63	15.42, 1.25
▪ 4 year (37–48 months)	2,456	15.11, 0.79	98.79, 2.18	15.73, 0.21
▪ 5 year (49–60 months)	2,842	17.59, 0.57	105.68, 1.97	15.90, 0.28

**Figure 1:**
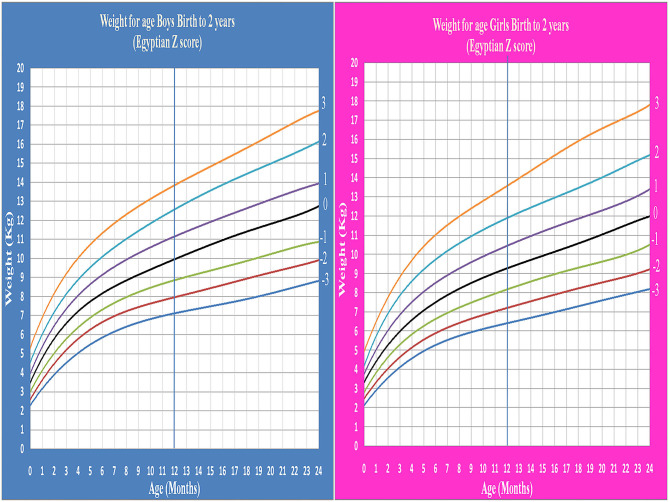
Egyptian Z score weight for age from birth to 2 years for boys & girls.

**Figure 2:**
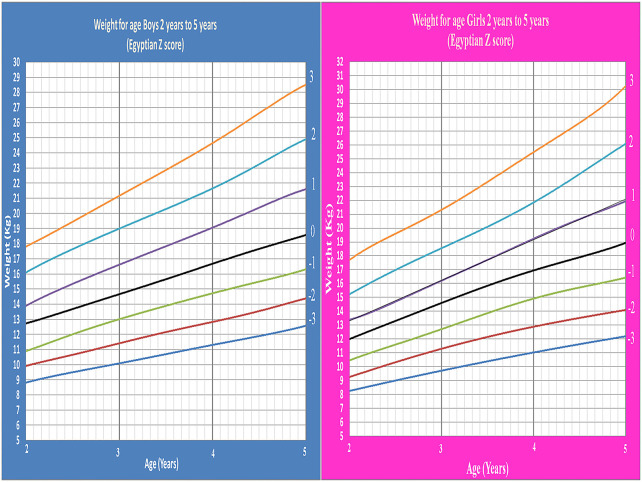
Egyptian Z score weight for age from 2 to 5 years for boys & girls.

**Figure 3:**
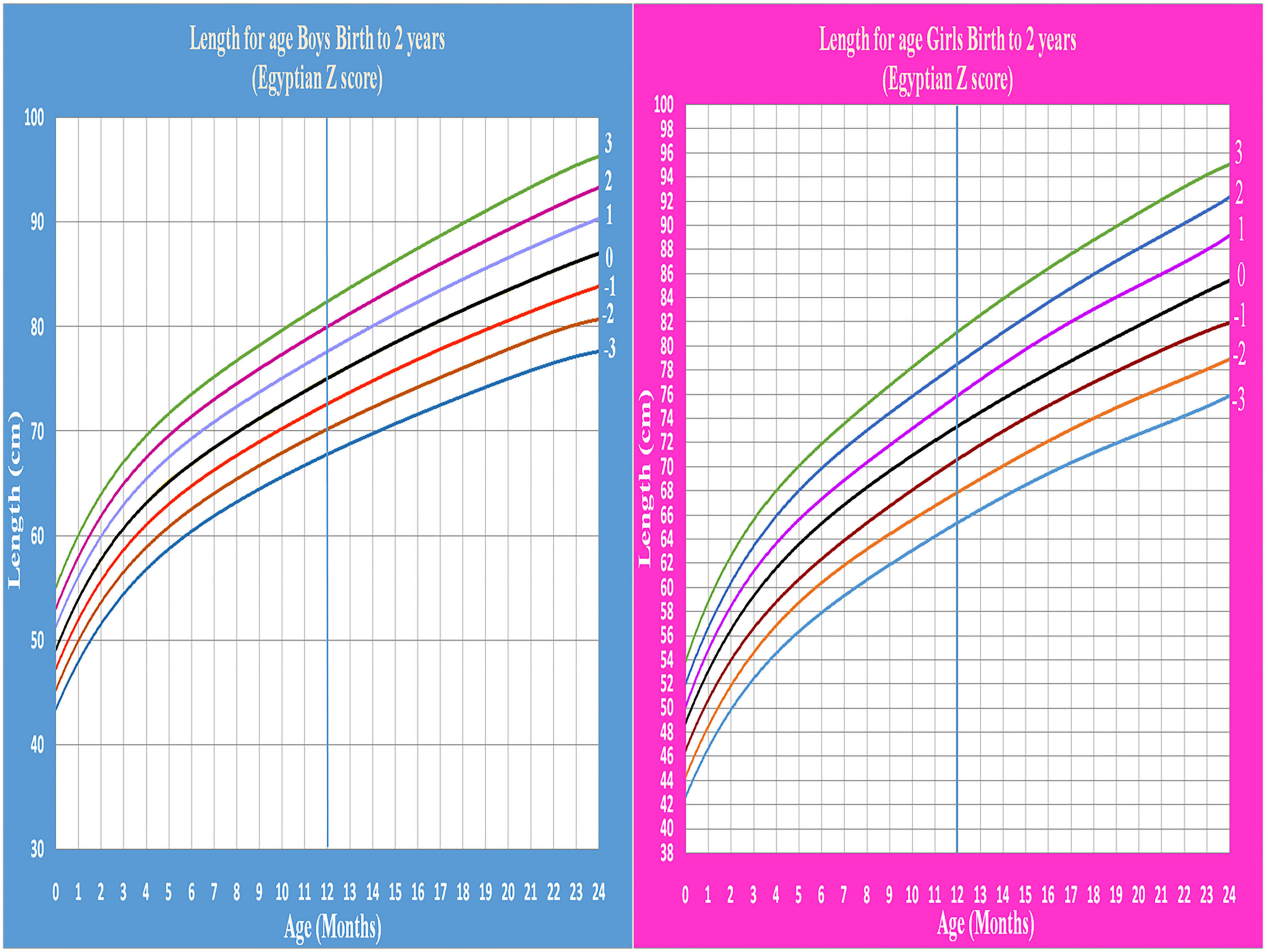
Egyptian Z score length for age from birth to 2 years for boys & girls.

**Figure 4:**
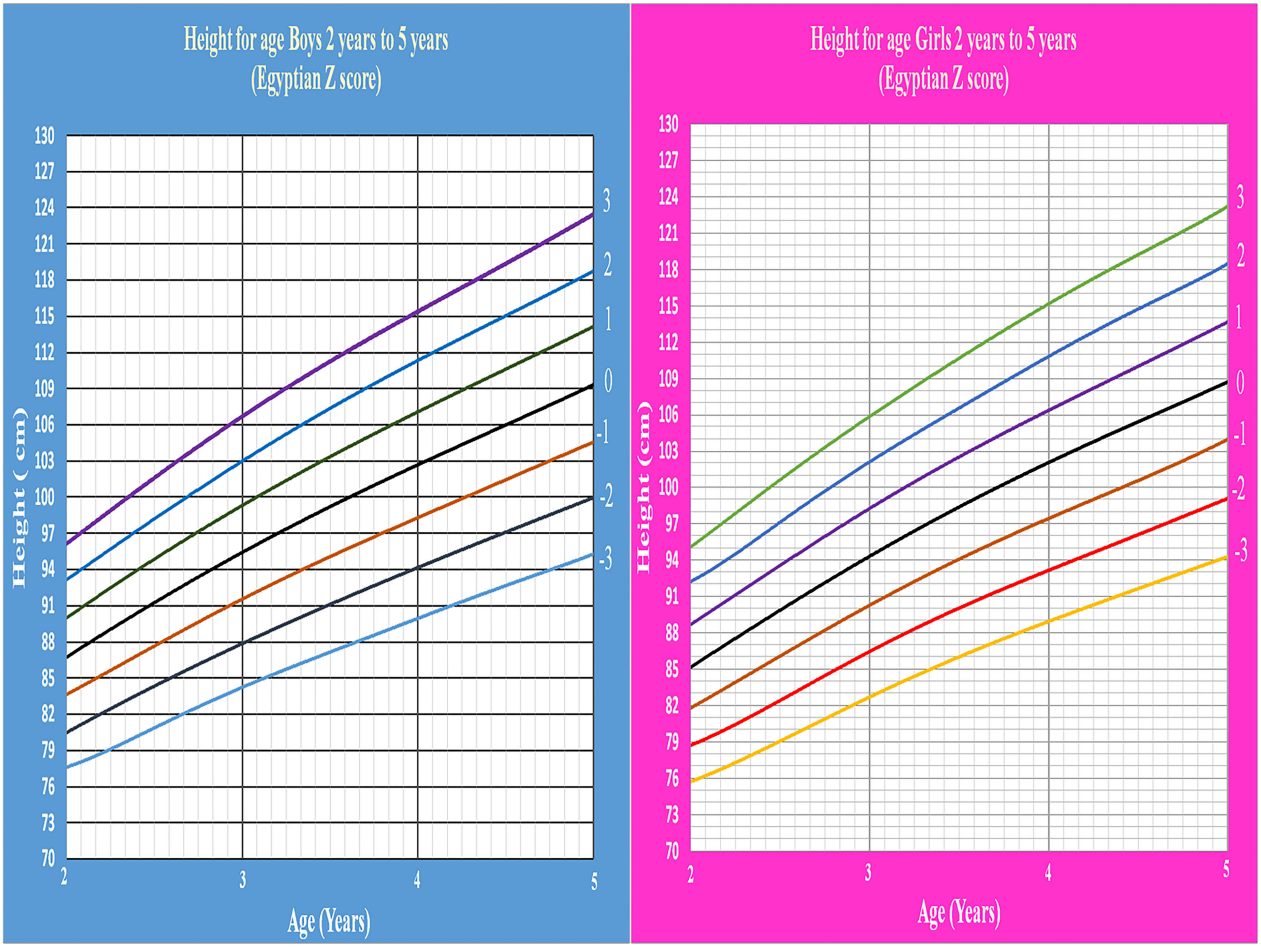
Egyptian Z score height for age from 2 to 5 years for boys & girls.

**Figure 5:**
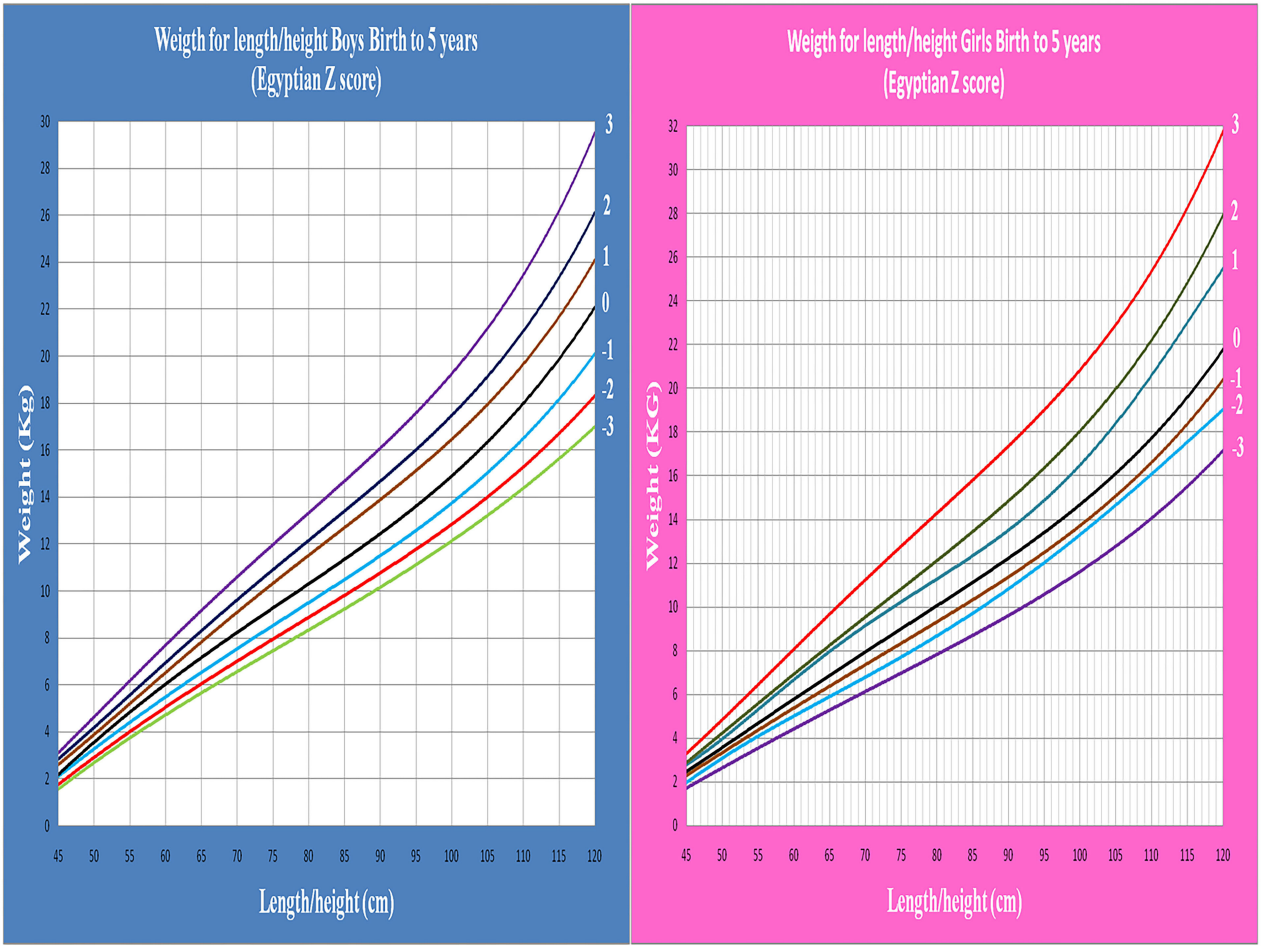
Egyptian Z score weight for length/height from birth to 5 years for boys & girls.

**Figure 6:**
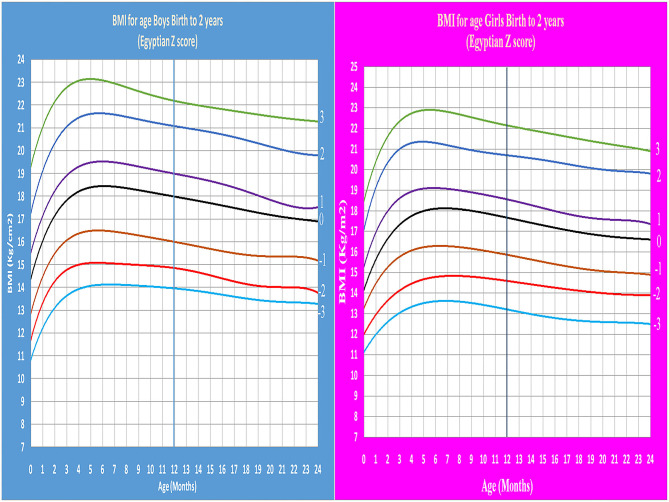
Egyptian Z score BMI for age from birth to 2 years for boys & girls.

**Figure 7:**
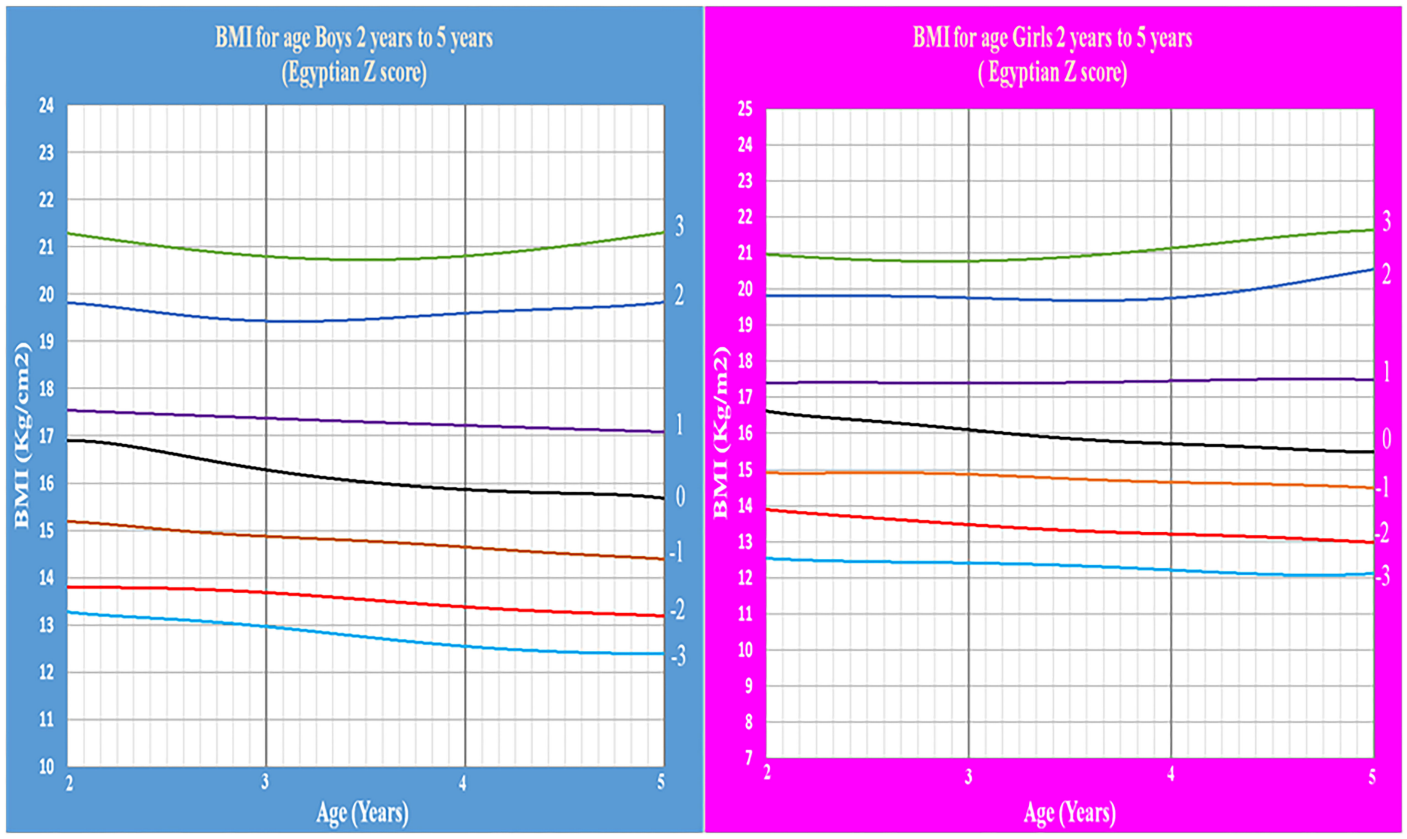
Egyptian Z score BMI for age from 2 to 5 years for boys & girls.

Age- and gender-specific Z score of Egyptian infants and children for weight for age, length/height for age, weight for length/height, and BMI for age were developed and smoothed by the LMS method (Table 2 and Figures 1–7).

**Table 2:** Egyptian L and S parameters and Z score weight-, Length/Height-, and BMI- for-age from birth up to 5 years.

	**Egyptian Z-score birth to 5 years**								
	**S**	**L**	**−3SD**	**−2SD**	**−1SD**	**Median**	**1SD**	**2SD**	**3SD**
**Boys**	**Weight-for-age**								
▪ 1 year (0–12 months)	0.12	0.19	5.36	6.06	6.75	7.63	8.50	9.50	10.64
▪ 2 year (13–24 months)	0.11	0.04	7.94	8.96	9.93	11.49	12.66	14.50	15.92
▪ 3 year (25–36 months)	0.11	−0.04	9.52	10.70	12.03	13.75	15.39	17.66	19.55
▪ 4 year(37–48 months)	0.12	−0.08	10.75	12.18	13.93	15.73	17.90	20.36	22.98
▪ 5 year (49–60 months)	0.56	−0.12	11.95	13.59	15.55	17.71	20.45	23.37	26.73
**Girls**									
▪ 1 year (0–12 months)	0.12	0.06	4.82	5.44	6.17	7.0	7.96	9.08	10.30
▪ 2 year (13–24 months)	0.12	−0.21	7.32	8.25	9.34	10.67	11.94	13.59	15.92
▪ 3 year (25–36 months)	0.13	−0.30	9.0	10.35	11.65	13.38	14.79	17.02	19.67
▪ 4 year(37–48 months)	0.13	−0.31	10.43	12.16	13.90	15.89	17.80	20.24	23.50
▪ 5 year (49–60 months)	0.14	−0.32	11.65	13.55	15.72	17.95	20.67	24.05	27.81
**Boys**	**Length/height-for-age**								
▪ 1 year (0–12 months)	2.32	1.0	58.73	60.86	63.06	65.23	67.60	69.72	71.88
▪ 2 year (13–24 months)	2.91	1.0	73.60	76.31	79.07	81.90	84.86	87.55	90.32
▪ 3 year (25–36 months)	3.45	1.0	81.15	84.60	87.96	91.50	95.17	98.53	101.95
▪ 4 year(37–48 months)	3.99	1.0	87.39	91.32	95.29	99.45	103.60	107.63	111.51
▪ 5 year (49–60 months)	4.49	1.0	92.87	97.32	101.71	106.28	110.88	115.34	111.71
**Girls**									
▪ 1 year (0–12 months)	2.35	1.0	56.71	58.99	61.26	63.78	66.03	68.33	70.56
▪ 2 year (13–24 months)	3.14	1.0	71.48	74.38	77.34	80.15	83.45	86.45	89.36
▪ 3 year (25–36 months)	3.67	1.0	79.57	83.07	86.62	90.22	94.06	97.60	101.17
▪ 4 year(37–48 months)	4.10	1.0	86.23	90.37	94.43	98.61	102.91	106.98	111.10
▪ 5 year (49–60 months)	4.60	1.0	91.95	96.47	101.12	105.69	110.48	115.04	119.61
**Boys**	**BMI**								
▪ 1 year (0–12 months)	0.08	−0.002	13.48	14.43	15.74	17.50	18.51	20.67	22.20
▪ 2 year (13–24 months)	0.07	−0.51	13.52	14.19	15.48	17.31	17.97	20.30	21.64
▪ 3 year (25–36 months)	0.08	−0.50	13.08	13.72	14.98	16.58	17.45	19.59	20.98
▪ 4 year(37–48 months)	0.08	−0.25	12.71	13.48	14.73	16.01	17.25	19.46	20.74
▪ 5 year (49–60 months)	0.08	−0.31	12.42	13.24	14.47	15.74	17.13	19.69	21.03
**Girls**									
▪ 1 year (0–12 months)	0.09	−0.09	13.0	14.20	15.60	17.27	18.30	21.41	21.95
▪ 2 year (13–24 months)	0.08	−0.54	12.70	14.12	15.22	16.96	17.79	20.16	21.45
▪ 3 year (25–36 months)	0.08	−0.61	12.42	13.64	14.89	16.30	17.40	19.75	20.81
▪ 4 year(37–48 months)	0.08	−0.61	12.27	13.30	14.73	15.85	17.40	19.70	20.93
▪ 5 year (49–60 months)	0.08	−0.61	12.07	13.10	14.54	15.59	17.45	20.11	21.42

The means of all age groups in the present study were compared with international WHO growth standard especially in the first years in which the comparison was made every 3 months. There were no significant differences for Mean values differences for height/length-, weight-, and BMI-for-age relative to the WHO references (*P* > 0.05) (Table 3).

**Table 3:** Weight for age, Length for age, and BMI for age of infants from birth up to 1 year relative to WHO growth references.

	**Boys**			**Girls**		
	**Mean ± SD**	**CI95%**	***t*-test/*P*-value**	**Mean ± SD**	**CI95%**	***t*-test/*P*-value**
**Weight (kg)-for-age**						
0–3 months						
• Egypt • WHO	5.15 ± 1.29 4.94 ± 1.31	3.51–6.49 3.34–6.17	0.23/0.823	4.94 ± 1.31 4.62 ± 1.14	3.34–6.37 3.25–5.70	0.36/0.827
3–6 months						
• Egypt • WHO	7.91 ± 0.24 7.48 ± 0.46	7.71–8.18 7.0–7.93	1.44/0.223	7.48 ± 0.46 7.02 ± 0.49	7.0–7.93 6.49–7.47	1.16/0.309
6–9 months						
• Egypt • WHO	8.77 ± 0.29 8.60 ± 0.30	8.47–9.05 8.29–8.90	1.23/0.230	8.60 ± 0.30 8.06 ± 0.32	8.29–8.90 7.75–8.39	1.36/0.186
9–12 months						
• Egypt • WHO	9.62 ± 0.23 9.40 ± 0.24	9.35–9.80 9.16–9.64	1.13/0.321	9.40 ± 0.24 8.93 ± 0.19	9.16–9.64 8.79–9.15	2.65/0.056
**Length (cm)-for-age**						
0–3 months						
• Egypt • WHO	55.38 ± 5.0 56.11 ± 4.97	49.15–60.76 49.88–60.67	0.20/0.842	54.31 ± 4.51 54.92 ± 4.59	48.70–59.15 49.14–59.11	0.19/0.856
3–6 months						
• Egypt • WHO	65.07 ± 1.82 65.80 ± 1.87	63.15–66.79 63.88–67.62	0.48/0.653	60.12 ± 7.49 63.95 ± 1.82	51.52–65.20 62.08–65.73	0.85/0.439
6–9 months						
• Egypt • WHO	66.47 ± 5.87 70.57 ± 1.40	59.86–71.11 69.16–71.96	0.79/0.436	68.10 ± 1.40 68.72 ± 1.42	66.69–69.50 67.28–70.14	0.76/0.455
9–12 months						
• Egypt • WHO	73.73 ± 1.27 74.52 ± 1.23	72.42–74.96 73.28–75.74	0.76/0.486	72.11 ± 1.16 72.75 ± 1.26	70.99–73.32 71.48–74.01	0.64/0.552
**BMI (kg/m**^**2**^**)**						
0–3 months						
• Egypt • WHO	15.97 ± 1.40 15.39 ± 1.55	14.10–17.10 13.40–16.80	0.55/0.598	15.32 ± 0.97 15.0 ± 1.33	14.0–16.10 13.33–16.25	0.38/0.714
3–6 months						
• Egypt • WHO	17.21 ± 0.12 17.26 ± 0.09	17.10–17.35 17.15–17.34	0.31/0.7708	16.46 ± 0.37 16.80 ± 0.12	16.20–16.90 16.67–16.90	1.47/0.214
6–9 months						
• Egypt • WHO	16.78 ± 0.68 17.25 ± 0.08	16.0–17.25 17.16–17.32	1.17/304	16.35 ± 0.31 16.82 ± 0.08	16.0–16.60 16.74–16.90	2.56/0.062
9–12 months						
• Egypt • WHO	17.23 ± 1.06 16.92 ± 0.12	16.0–17.90 16.79–17.04	0.49/0.644	16.63 ± 0.55 16.48 ± 0.13	16.0–17.0 16.35–16.61	0.64/0.695

*Test of significance: Un-paired t-test*.

**Figure 8:**
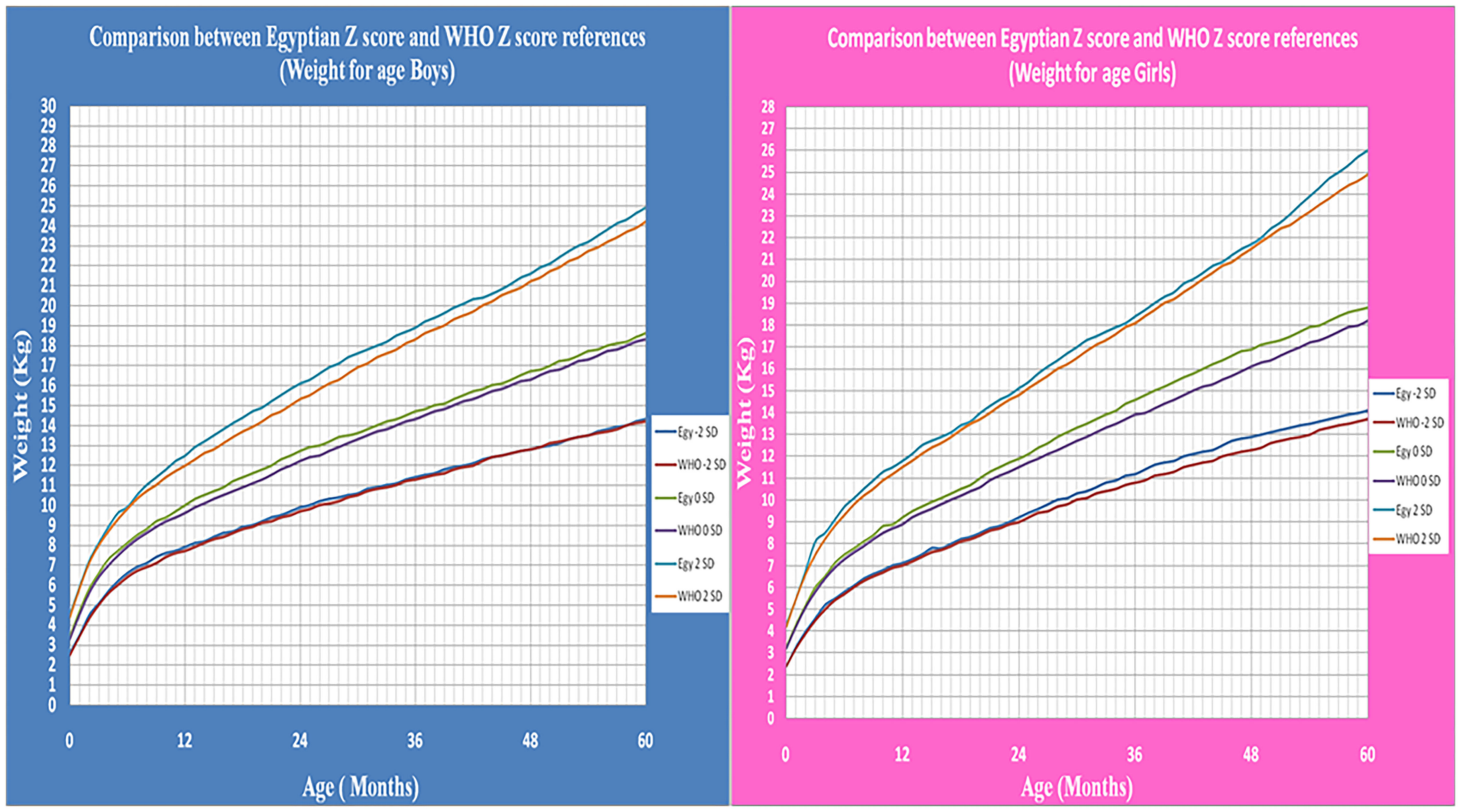
The comparison between Egyptian Z score and WHO Z score references value from birth up to 5 years (weight for age in boys & girls).

**Figure 9:**
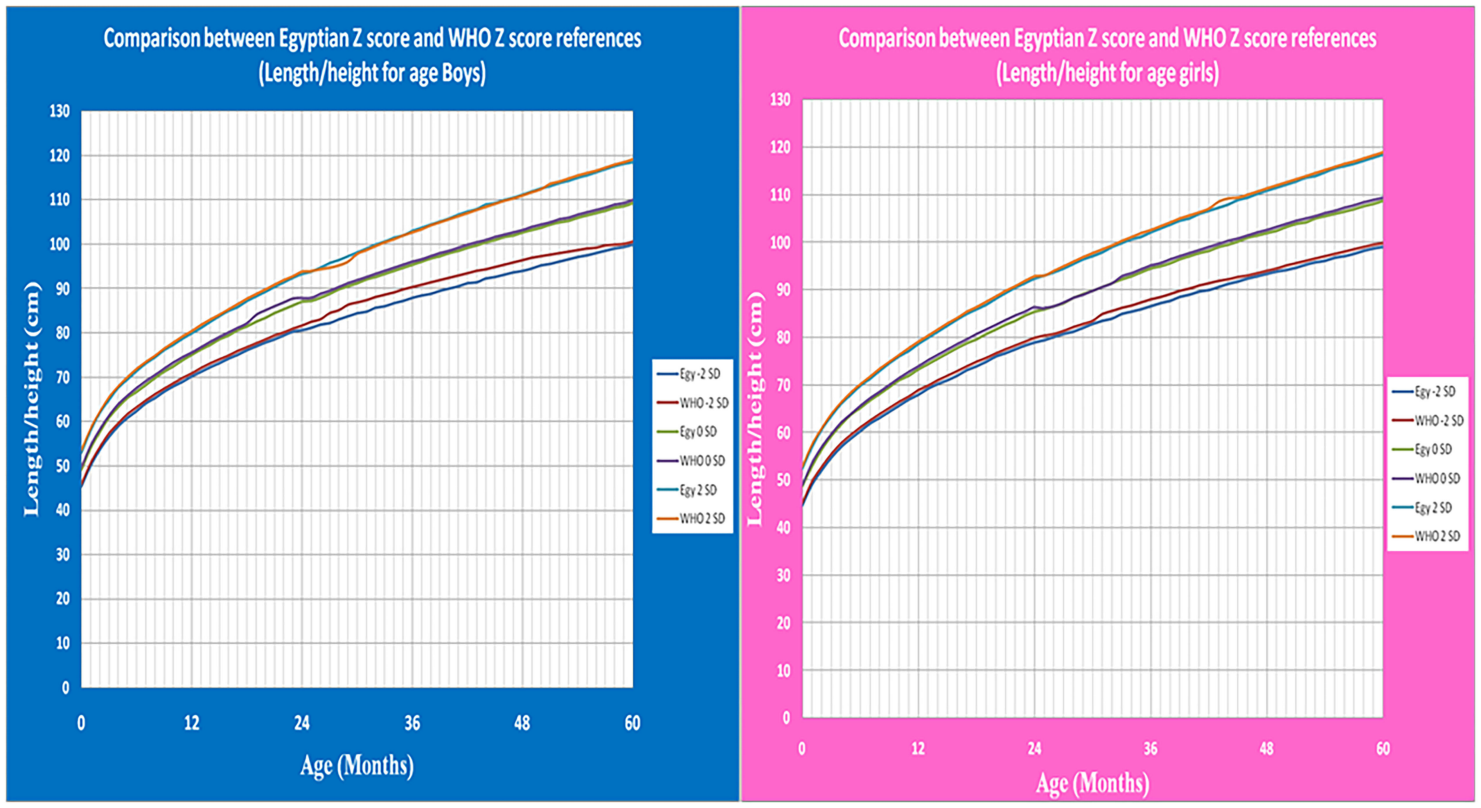
The comparison between Egyptian Z score and WHO Z score references value from birth up to 5 years (Length/height for age in boys & girls).

The means of all age groups in the present study were compared with international WHO growth standard. There were no significant differences for Mean values differences for height/length, weight, and BMI-for-age relative to the WHO references (*P* > 0.05). Difference in means values for weight for length/height relative to the WHO references was not significant (boys: 11.28 ± 5.35, 95% CI 10.47–12.15; girls: 11.07 ± 5.28, 95% CI 11.42 ± 5.65; total: 11.18 ± 5.31, 95% CI−11.40 ± 5.59) (Table 4 and Figures 8, 9).

**Table 4:** Weight for age, length/height for age, BMI for age, and weight for length/height of children from birth up to 5 years relative to WHO growth standards.

	**Boys**			**Girls**		
	**Mean ± SD**	**CI95%**	***t*-test/*P*-value**	**Mean ± SD**	**CI95%**	***t*-test/*P*-value**
**Weight (kg)-for-age**						
1 year (0–12 months)						
• Egypt • WHO	7.45 ± 1.97 7.21 ± 1.93	6.32–8.46 6.09–8.17	0.29/0.769	6.78 ± 1.82 6.66 ± 1.76	5.73–7.74 5.65–7.54	0.15/0.876
2 year (13–24 months)						
• Egypt • WHO	11.19 ± 0.81 10.82 ± 0.75	10.73–11.66 10.38–11.23	1.17/0.255	10.40 ± 0.80 10.12 ± 0.76	9.94–10.86 9.68–10.53	0.87/0.389
3 year (25–36 months)						
• Egypt • WHO	13.53 ± 0.59 13.19 ± 0.66	13.18–13.86 12.84–13.54	1.34/0.191	13.12 ± 0.80 12.59 ± 0.71	12.65–13.57 12.21–12.98	1.73/0.098
4 year (37–48 months)						
• Egypt • WHO	15.56 ± 0.58 15.26 ± 0.60	15.26–15.90 14.92–15.60	1.23/0.230	15.28 ± 0.79 14.87 ± 0.66	14.84–15.72 14.49–15.25	1.36/0.186
5 year (49–60 months)						
• Egypt • WHO	17.60 ± 0.61 17.34 ± 0.64	17.25–17.91 16.99–17.70	1.03/0.310	17.45 ± 0.84 17.15 ± 0.69	17.04–17.92 16.77–17.53	1.16/0.257
**Length/height (cm)-for-age**						
1 year (0–12 months)						
• Egypt • WHO	64.16 ± 7.93 65.11 ± 7.72	59.60–68.33 60.54–69.12	0.30/0.467	62.08 ± 8.07 63.49 ± 7.38	57.30–66.37 59.18–67.32	0.44/0.658
2 year (13–24 months)						
• Egypt • WHO	80.86 ± 3.65 81.59 ± 3.66	78.80–82.95 79.53–83.57	0.48/0.630	79.11 ± 3.65 80.02 ± 3.76	77.07–81.15 77.91–82.06	0.60/0.553
3 year (25–36 months)						
• Egypt • WHO	90.76 ± 2.63 91.50 ± 2.62	89.26–92.21 90.09–92.91	0.69/0.496	91.06 ± 3.72 90.24 ± 2.73	88.94–93.08 88.78–91.71	0.61/0.545
4 year (37–48 months)						
• Egypt • WHO	98.79 ± 2.18 99.49 ± 2.19	97.52–99.98 98.26–100.66	0.79/0.436	97.94 ± 2.33 98.67 ± 2.32	96.59–99.22 97.36–99.91	0.76/0.455
5 year (49–60 months)						
• Egypt • WHO	105.96 ± 2.14 106.65 ± 2.15	104.81–107.12 105.54–107.84	0.82/0.418	105.44 ± 2.16 106.13 ± 2.17	104.27–106.60 105.00–107.33	0.81/0.423
**BMI (kg/m**^**2**^**)**						
1 year (0–12 months)						
• Egypt • WHO	17.44 ± 1.11 16.52 ± 1.20	16.73–17.97 15.77–17.09	1.94/0.065	17.32 ± 2.55 16.15 ± 1.10	16.16–18.72 15.43–16.67	1.46/0.157
2 year (13–24 months)						
• Egypt • WHO	16.52 ± 0.42 16.22 ± 0.33	16.25–16.73 16.04–16.41	1.88/0.073	16.16 ± 0.60 15.81 ± 0.30	15.81–16.46 15.65–15.98	1.74/0.095
3 year (25–36 months)						
• Egypt • WHO	16.01 ± 0.47 15.79 ± 0.11	15.74–16.25 15.73–15.86	1.58/0.128	15.87 ± 1.22 15.52 ± 0.08	15.13–16.42 15.47–15.56	1.0/0.328
4 year (37–48 months)						
• Egypt • WHO	15.57 ± 0.28 15.46 ± 0.07	15.41–15.73 15.42–15.51	1.33/207	15.38 ± 0.25 15.32 ± 0.04	15.25–15.54 15.30–15.34	0.86/0.406
5 year (49–60 months)						
• Egypt • WHO	15.38 ± 0.24 15.25 ± 0.05	15.25–15.52 15.23–15.28	1.87/0.085	15.41 ± 0.28 15.25 ± 0.01	15.28–15.56 15.25–15.26	2.06/0.062
**Weight for length/height**						
• Egypt • WHO	11.28 ± 5.35 11.39 ± 5.54	10.47–12.15 10.55–12.36	0.17/0.863	11.07 ± 5.28 11.42 ± 5.65	10.28–11.93 10.44–12.29	0.54/0.586

*Test of significance: Un-paired t-test*.

**Figure 10:**
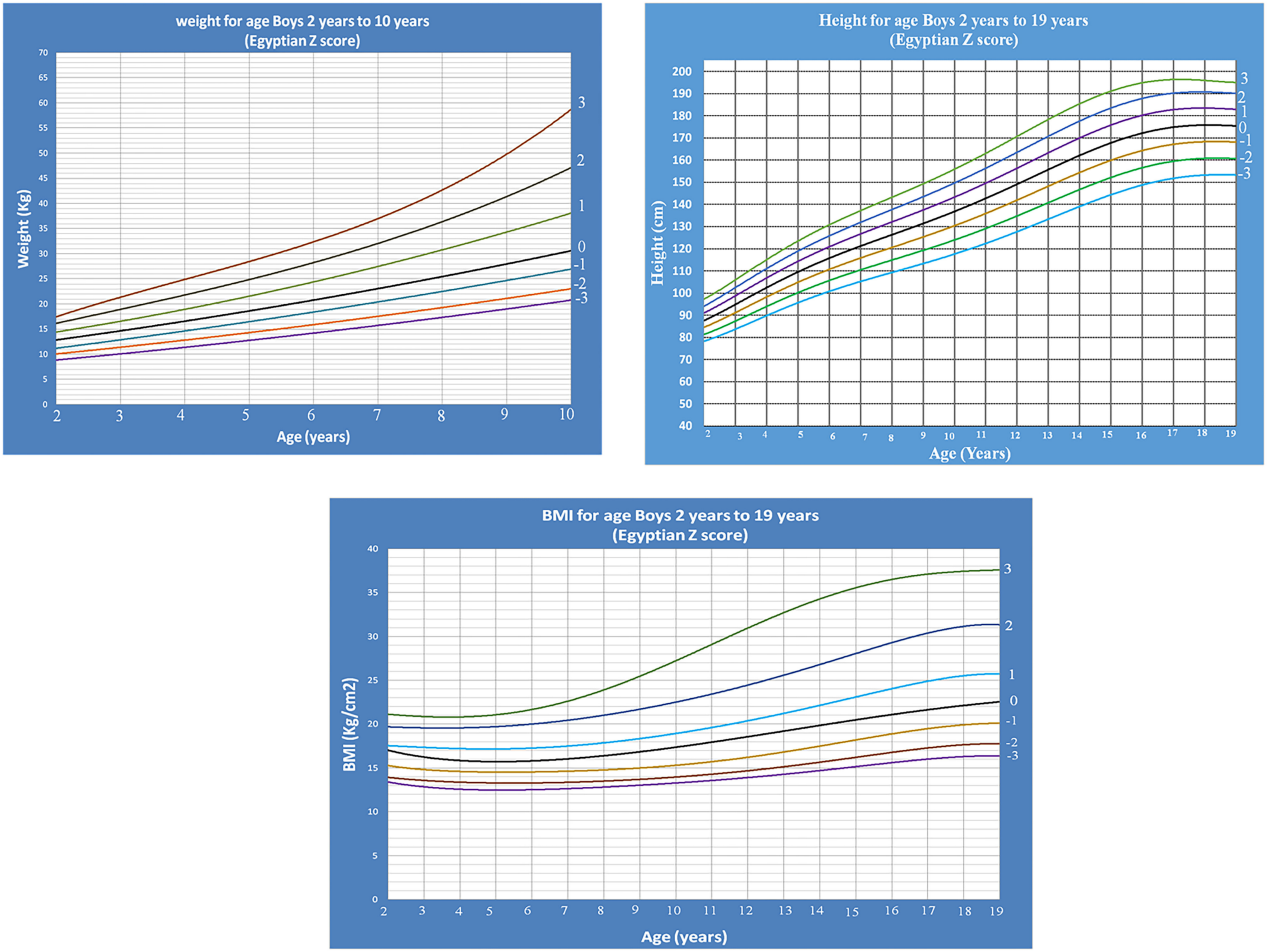
Egyptian Z score weight-, height-, BMI- for age from 2 to 19 years for boys.

**Figure 11:**
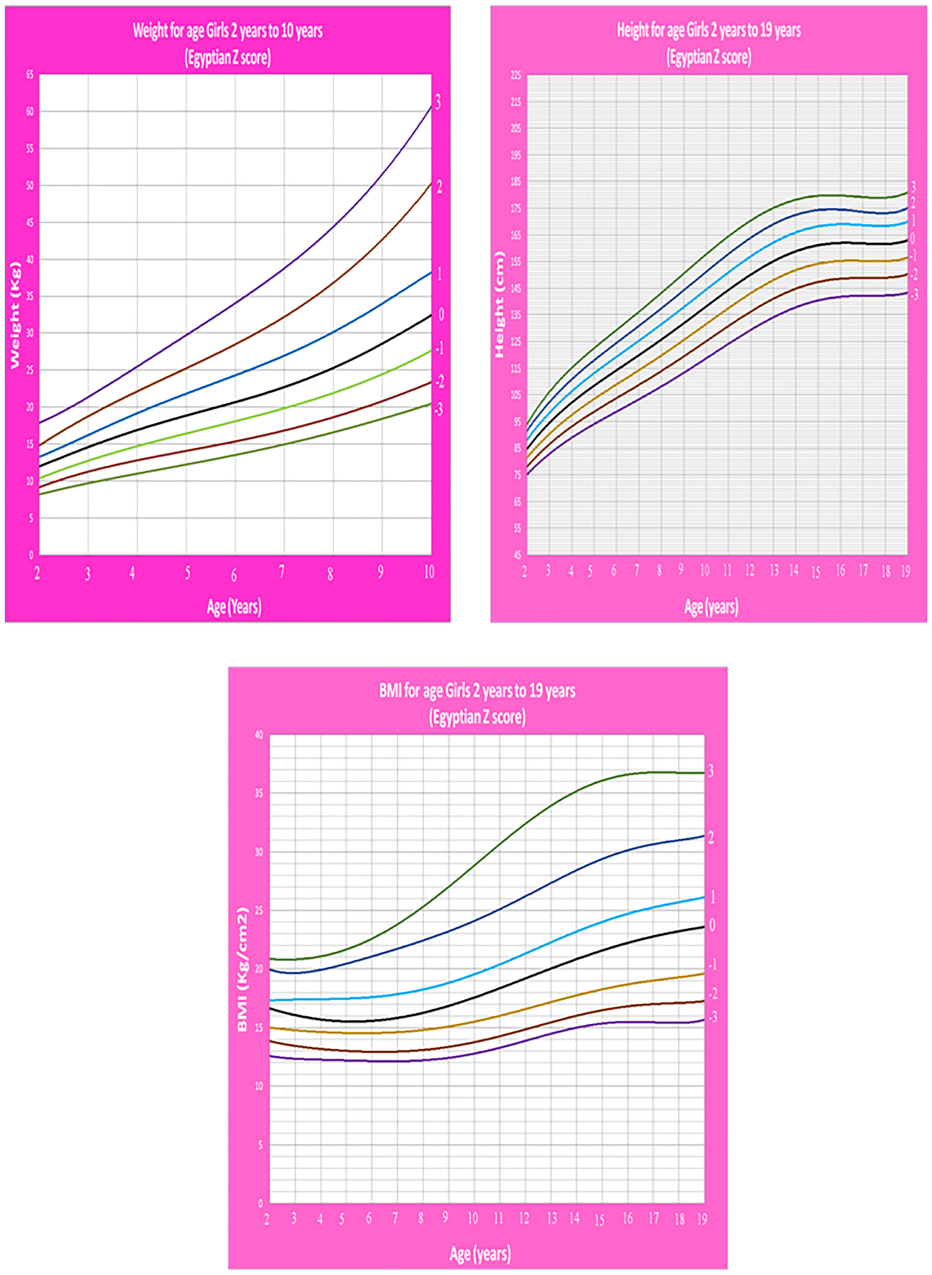
Egyptian Z score weight-, height-, BMI- for age from 2 to 19 years for girls.

Age- and gender-specific Z score growth charts from 2 to 19 years [by combining our data of the present study with the data of our previous study (14)] of Egyptian children weight for age, height for age, and BMI for age were developed and smoothed by the LMS method (Figures 10, 11).

## Discussion

Z score growth charts are designed as x axis (age in months/years) and y axis (according to the measured parameter). In our charts, the x axis is divided into 12 equal parts (months per year) as WHO growth standard (7), Centers for Disease Control and Prevention (CDC) (24) and Indian charts (25), unlike growth charts in the United Kingdom, decimal ages were used where each years is divided to 10 parts (26).

Weight for age and length/height for age charts show a rapid linear increase in the first 2 years of life then continue at a slower rate. This agrees with Karlberg's infancy-childhood-puberty (ICP) model which reported that about two-thirds of normal children are reported to “shift centiles” for linear growth during the first 12 to 18 months of life (27). Growth is more stable during the third year of life. After 6 months of age, 90% of healthy infants show a height increase of more than 0.2 SD per 3 months. The rate of growth between 4 and 7 years of age is correlated with the velocity during the second year of life (28).

In clinical settings, BMI for age and weight for stature (length/height) are ratios of weight relative to stature. Weight for stature in preschool children is used to know and screen for nutritional status (underweight and overweight) (29, 30). Z score growth charts of weight for stature were measured till age of 5 years. This is because BMI for age and weight for stature correlates evenly with total body fat for children below 5 years, however BMI for age correlates better after this age. This is concluded from a study by CDC (31) which compared BMI for age and weight for stature with fatness measured by dual energy x-ray absorptiometry (DXA), a direct measure of adiposity (32).

There is a difference in shape between weight for stature and the BMI for age growth curves. BMI for age starts to decrease after about the first year and continue to decrease till the age of 5 years. After 5 years the curve starts to gradually increase till reach the adolescent which is similar to adulthood. This is a physiological phenomenon known as “adiposity” rebound (33–35).

Growth chart are divided into two types. The first is the growth standards, with the data collection from children with optimal nutrition and health. They provide a prescriptive tool of how a child should grow. The second is growth references, which are a descriptive method of how the population with the best possible nutrition and health are growing rather than how they should be growing (10).

This study presents the first national reference to describe the growth of normal Egyptian preschool children using the L, M, and S parameters. Weight, length/height, and body mass index corresponding to age, also weight for length/height Z score growth parameters were created. WHO growth standards is widely used in Egypt, so we had to follow its approach and methodology when starting our study in order for us to make a comparison to test the significance between our results and their outcomes. Therefore, our study was divided with the same methodology like WHO. The age of five years was taken into account as a breaking point similar to the WHO growth charts that was considered as a standard charts in preschool children (7, 36). Although the growth curves was extended only to 60 months, data were collected until 71 months of age and all data was used to construct the curves to avoid the right-edge effect, so extending our study from 5 to 6 years stabilized the leverage function within the target age reference interval from birth up to 5 years (16).

So the current results are consistent with our study published in 2020 on Egyptian school children and adolescents aged 5–19 years and considered complementary to it as both studies together make a reference for growth and nutritional status among Egyptian infants, children and adolescents from birth up to 19 years (14).

The design of our study largely fulfills the criteria suggested by Waterlow and WHO which required the reference population to be well-nourished, the sampling procedure clearly defined and reproducible, the sample size is adequate, the measurements are relevant and of good quality and the data are adequately processed (37). In view of the known variations in growth in different populations, the availability of this reference which is based on a national representative sample is important for accurate estimation of the nutritional status and growth of Egyptian infants and children. After comparison of weight, length/height, and BMI values of both sexes of the children who participated in the present study with the WHO values, our references of Egyptian preschool children showed slight differences from the standard values of WHO as we found that Egyptian children were heavier and shorter than children in WHO charts but with no significant differences (*P* > 0.05) (7). This supports the hypothesis that the WHO Multicenter Growth Reference Study was expected to provide an international standard that represents a description of physiological growth for all children and was also expected to be used to assess children everywhere (9). Also, our study agreed with a studies done on Polish children when compared with WHO reference value (38–40). This Compatibility with WHO references disagrees with Saudi national Z-score charts 2017, which found downward displacement of the Saudi charts by −2 SD from that of the WHO charts. The consequence of this finding is considerably important clinically as leads to overestimation of the prevalence of underweight and stunting (41).

Comparing the results of a pervious study conducted in Egypt on 2002 with the results of the current study was not possible, since either the growth charts were given without base line tables or the age grouping was every 3 or 5 months, unlike the present study which was done for each single month (13). Therefore, it is not possible to determine the secular trend of children at this stage; also the previous study was based on local data of small sample size that collected from only one governorate in Egypt using percentile methods. In contrast to the present study in which the data was collected from eight governorates in order to be able to represent the whole Egypt, the appropriate sample size was taken in account and the study was done by using Z score method to be more accurate.

The method we used in our study to analyze the data was originally developed by Cole who summarized growth charts as LMS parameters to obtain the exact percentiles and/ or Z score to include the whole deviation from the growth references (42). As a result, several studies have been conducted in favor of using of LMS analysis to develop the age and gender specific smoothed growth reference charts (43–45).

The Z score is more accurate than percentiles as children below the third percentile or more than 97 percentile are considered abnormal, but when using a Z score there can be an additional subdivision into −2 to −3 or less and +2 to + 3 or more SD, respectively. This allows for a better evaluation of growth abnormalities and thus a better follow-up of effective treatment measures (46).

The advantages of our growth references are that they are true representative of the existing growth pattern of children and allow us to study the secular trend in terms of height, weight, and BMI. The downside of reference curves is that they need to be updated at least once in a decade and in modern times where obesity is on the rise, children who are overweight are more likely to be identified as normal. As the growth pattern changes with time, it is recommended to update the references regularly.

The possibility of using our Egyptian Z score reference parameters in describing growth and nutritional disorders in Egyptian preschool children is now available, so a child whose weight for age is below −2 is considered underweight and below −3 is considered severely underweight. Wasting is diagnosed when weight for length/height below −2 SD. Regarding length for age, a child who's below −2 is considered stunted and below −3 is considered severely stunted in accordance with the definition of WHO (17). The availability of Egyptian LMS parameters and Z-scores references allows the application of the recent WHO definitions of overweight (BMI >+1 SD), obesity (BMI >+2 SD), and thinness (BMI < -2 SD).The growth reference values need to be re-estimated every 5–10 years for populations with suspected large secular changes (47).

## Conclusion

The availability of this national Z-score reference for Egyptian children from birth up to 5 years will facilitate more accurate assessment of growth and nutritional status of Egyptian children under different clinical conditions rather than using references from other populations. Also we recommend updating growth data every 10 years, maximum, to follow the national secular trend in the growth.

## Data Availability Statement

The raw data supporting the conclusions of this article will be made available by the authors, without undue reservation.

## Ethics Statement

The studies involving human participants were reviewed and approved by Ethical approval (ID: 190118; Ped) was obtained from institutional research board in Menoufia Faculty of Medicine work in accordance with the Declaration of Helsinki. Written informed consent to participate in this study was provided by the participants' legal guardian/next of kin.

## Author Contributions

Idea, design and data interpretation: AES, ZO, AE-B, and WB. Participant enrolment and data collection: ZO, AE-B, MAE-F, AA, AK, HR, GB, AGS, WG, WB, SA, AAS, and NF. Manuscript writing: ZK, HH, AE-B, ZO, and WB. Statistical analysis: AE-B and ZK. Manuscript revision: AES, ZO, FE-G, DA, MS, AE-B, WB, and ZK. All authors contributed to the article and approved the submitted version.

## Conflict of Interest

The authors declare that the research was conducted in the absence of any commercial or financial relationships that could be construed as a potential conflict of interest.
