# Supplementary material for: Development of LMS and Z Score Growth References for Egyptian Children From Birth Up to 5 Years
Source: Front Pediatr. 2021 Jan 18;8:598499. doi: 10.3389/fped.2020.598499 (PMC7849193; doi:10.3389/fped.2020.598499)
Supplement: Supplementary file 1 [file Data_Sheet_1.PDF]

Table (1): shows Egyptian L, M and S parameters and Z score weight for age for boys from birth to 5 years.

Weight-for-age BOYS Egyptian Z-score Birth to 5 Years											
Y:M	M	Mean	S	L	-3SD	-2SD	-1SD	Median	1SD	2SD	3SD
00:00	0	3.5125	0.15633	0.4589	2.2	2.6	3	3.4	3.9	4.5	5.2
00:01	1	4.7956	0.14568	0.3255	3.1	3.5	4.1	4.7	5.2	5.9	6.8
00:02	2	5.8218	0.13587	0.2658	3.9	4.5	5	5.8	6.5	7.2	8.2
00:03	3	6.4953	0.12985	0.1999	4.5	5.1	5.8	6.6	7.4	8.1	9.2
00:04	4	7.8563	0.11999	0.1874	5	5.7	6.3	7.3	7.9	8.9	9.9
00:05	5	7.71598	0.11352	0.1789	5.4	6.2	6.8	7.7	8.6	9.6	10.7
00:06	6	8.1854	0.11036	0.1654	5.8	6.6	7.2	8.1	9	9.9	11.2
00:07	7	8.4762	0.10947	0.1541	6.1	6.9	7.6	8.5	9.5	10.5	11.7
00:08	8	8.7952	0.10936	0.1412	6.4	7.1	8	8.8	9.9	11	12.4
00:09	9	9.0568	0.10925	0.1369	6.6	7.4	8.2	9.2	10.2	11.4	12.7
00:10	10	9.3598	0.10901	0.1258	6.8	7.6	8.4	9.4	10.5	11.8	13.1
00:11	11	9.7254	0.10987	0.1123	6.9	7.7	8.6	9.7	10.9	12.2	13.5
01:00	12	9.8035	0.10999	0.1023	7	7.9	8.8	10	11.1	12.5	13.8
01:01	13	10.2486	0.11009	0.0999	7.2	8.1	9	10.3	11.4	12.9	14.1
01:02	14	10.3852	0.11125	0.0852	7.3	8.2	9.2	10.5	11.6	13.2	14.4
01:03	15	10.8563	0.11189	0.0789	7.4	8.4	9.3	10.7	11.8	13.5	14.7
01:04	16	10.8965	0.11201	0.0654	7.6	8.6	9.5	10.9	12.1	13.8	15.1
01:05	17	11.0256	0.11255	0.0569	7.7	8.7	9.7	11.2	12.3	14.1	15.5
01:06	18	11.2596	0.11289	0.0456	7.8	8.9	9.8	11.4	12.6	14.4	15.8
01:07	19	11.5231	0.11305	0.0325	8	9	10	11.6	12.8	14.7	16.1
01:08	20	11.726	0.11325	0.0258	8.1	9.2	10.2	11.8	13	14.9	16.4
01:09	21	11.958	0.11375	0.0145	8.3	9.4	10.4	12	13.3	15.2	16.8
01:10	22	12.2159	0.11389	0.0048	8.5	9.5	10.5	12.3	13.5	15.5	17.1
01:11	23	12.4652	0.11401	0.0039	8.6	9.7	10.7	12.5	13.7	15.8	17.4
02:00	24	12.6258	0.11425	-0.0089	8.8	9.9	10.9	12.7	13.9	16.1	17.7
02:01	25	12.8123	0.11521	-0.0189	8.9	10	11	12.9	14.1	16.3	18
02:02	26	12.8953	0.11565	-0.0225	9	10.2	11.2	13	14.4	16.6	18.3
02:03	27	13.125	0.11622	-0.0265	9.1	10.3	11.4	13.2	14.6	16.9	18.6
02:04	28	13.3852	0.11699	-0.0299	9.3	10.4	11.6	13.4	14.9	17.1	18.8
02:05	29	13.5012	0.11714	-0.0321	9.4	10.5	11.8	13.5	15.1	17.4	19.1
02:06	30	13.6021	0.11798	-0.0356	9.5	10.6	12	13.6	15.3	17.6	19.4
02:07	31	13.7202	0.11813	-0.0455	9.6	10.8	12.1	13.8	15.5	17.8	19.7
02:08	32	13.963	0.11879	-0.0499	9.7	10.9	12.3	14	15.7	18	20
02:09	33	14.1026	0.11914	-0.0525	9.8	11	12.5	14.2	15.9	18.2	20.3
02:10	34	14.2035	0.11954	-0.0565	9.9	11.1	12.7	14.3	16.2	18.5	20.5
02:11	35	14.4962	0.11999	-0.0599	10	11.3	12.8	14.5	16.4	18.7	20.8
03:00	36	14.6322	0.12007	-0.0625	10.1	11.4	13	14.7	16.6	18.9	21.1
03:01	37	14.7521	0.12078	-0.0655	10.2	11.5	13.1	14.8	16.8	19.2	21.4
03:02	38	14.9527	0.12114	-0.0699	10.3	11.6	13.3	15	17	19.4	21.7
03:03	39	15.9258	0.12165	-0.0735	10.4	11.8	13.4	15.1	17.2	19.6	22

03:04	40	15.2017	0.12255	-0.0755	10.5	11.9	13.6	15.3	17.4	19.9	22.3
03:05	41	15.4203	0.12289	-0.0799	10.6	12	13.8	15.5	17.6	20.1	22.5
03:06	42	15.6207	0.12315	-0.0825	10.7	12.1	13.9	15.7	17.8	20.3	22.8
03:07	43	15.6028	0.12366	-0.0845	10.8	12.3	14	15.8	18	20.4	23.1
03:08	44	15.9852	0.12478	-0.0887	10.9	12.4	14.1	16	18.2	20.6	23.4
03:09	45	16.0258	0.12489	-0.0898	11	12.5	14.3	16.1	18.4	20.8	23.7
03:10	46	16.2152	0.12512	-0.0914	11.1	12.6	14.4	16.3	18.6	21.1	24
03:11	47	16.4205	0.12587	-0.0936	11.2	12.7	14.6	16.5	18.8	21.4	24.3
04:00	48	16.6652	0.12623	-0.0945	11.3	12.8	14.7	16.7	19.1	21.6	24.6
04:01	49	16.785	0.12699	-0.1035	11.4	12.9	14.8	16.8	19.3	21.9	24.9
04:02	50	16.9857	0.12755	-0.1089	11.5	13	15	17	19.5	22.1	25.2
04:03	51	17.1953	0.12896	-0.1111	11.6	13.1	15.1	17.2	19.7	22.4	25.6
04:04	52	17.2038	0.12987	-0.1125	11.7	13.3	15.2	17.3	19.9	22.7	25.9
04:05	53	17.4035	0.12999	-0.1145	11.8	13.4	15.4	17.5	20.1	23	26.3
04:06	54	17.6205	0.13005	-0.1201	11.9	13.5	15.5	17.7	20.3	23.2	26.6
04:07	55	17.8632	0.13089	-0.1289	12	13.7	15.6	17.8	20.6	23.5	26.9
04:08	56	17.9025	0.13147	-0.1314	12.1	13.8	15.7	18	20.8	23.8	27.2
04:09	57	18.0935	0.13258	-0.1368	12.2	13.9	15.9	18.1	21	24.1	27.6
04:10	58	18.1852	0.13354	-0.1389	12.3	14	16	18.2	21.2	24.3	27.9
04:11	59	18.3028	0.13458	-0.1425	12.4	14.2	16.1	18.4	21.4	24.6	28.2
05:00	60	18.6025	0.13589	-0.1445	12.5	14.3	16.3	18.6	21.6	24.9	28.5

Table (2): shows Egyptian L, M and S parameters and Z score weight for age for girls from birth to 5 years.

Weight-for-age GIRLS Egyptian Z-score birth to 5 Years											
Y:M	M	Mean	S	L	-3SD	-2SD	-1SD	Median	1SD	2SD	3SD
00:00	0	3.2584	0.15271	0.4558	2.1	2.4	2.9	3.3	3.8	4.3	4.9
00:01	1	4.20158	0.14789	0.3698	2.8	3.3	3.6	4.3	4.9	5.5	6.3
00:02	2	5.12587	0.13698	0.2584	3.5	4	4.7	5.2	5.9	6.8	7.8
00:03	3	5.90258	0.13111	0.1458	4.1	4.6	5.3	6	6.8	8.1	8.8
00:04	4	6.49856	0.12789	0.0587	4.5	5.2	5.8	6.5	7.6	8.5	9.7
00:05	5	7.09852	0.12655	-0.0099	4.9	5.5	6.2	7.1	8	9.1	10.4
00:06	6	7.4785	0.12555	-0.0122	5.2	5.8	6.6	7.5	8.5	9.7	11
00:07	7	7.75215	0.12501	-0.0256	5.4	6.1	6.9	7.8	8.8	10.1	11.4
00:08	8	8.05633	0.12499	-0.0596	5.7	6.4	7.1	8.1	9.3	10.5	11.8
00:09	9	8.39851	0.12355	-0.0658	5.9	6.6	7.4	8.4	9.5	10.9	12.4
00:10	10	8.79532	0.12255	-0.0789	6	6.8	7.8	8.8	9.9	11.3	12.9
00:11	11	8.8523	0.12245	-0.1099	6.2	7	7.9	8.9	10.2	11.5	13.1
01:00	12	9.15422	0.12201	-0.1103	6.4	7.1	8.1	9.2	10.4	11.8	13.5
01:01	13	9.45832	0.12198	-0.1214	6.5	7.3	8.3	9.5	10.6	12.1	13.9
01:02	14	9.62015	0.12205	-0.1658	6.6	7.5	8.6	9.7	10.9	12.5	14.2
01:03	15	9.8521	0.12225	-0.1789	6.8	7.8	8.7	9.9	11.2	12.7	14.8
01:04	16	10.0958	0.12265	-0.2035	6.9	7.8	8.9	10.1	11.4	12.9	15.1
01:05	17	10.2587	0.12285	-0.2125	7.1	8	9.1	10.3	11.6	13.1	15.5
01:06	18	10.4359	0.12301	-0.2258	7.3	8.2	9.3	10.5	11.8	13.4	15.9
01:07	19	10.6582	0.12325	-0.2288	7.4	8.3	9.4	10.7	12	13.6	16.2
01:08	20	10.9521	0.12365	-0.2301	7.6	8.5	9.5	11	12.3	14	16.5
01:09	21	11.2159	0.12455	-0.2321	7.7	8.7	9.7	11.3	12.5	14.3	16.8
01:10	22	11.4588	0.12498	-0.2457	7.9	8.8	10	11.5	12.7	14.6	17.1
01:11	23	11.6522	0.12502	-0.2596	8	9	10.2	11.7	13	14.8	17.4
02:00	24	11.8563	0.12525	-0.2699	8.1	9.2	10.4	11.9	13.3	15.1	17.7
02:01	25	12.0985	0.12555	-0.2879	8.3	9.4	10.6	12.1	13.5	15.4	18
02:02	26	12.3254	0.12575	-0.2988	8.4	9.6	10.8	12.4	13.7	15.8	18.3
02:03	27	12.5632	0.12625	-0.3009	8.6	9.8	11	12.6	13.9	16.1	18.7
02:04	28	12.8965	0.12655	-0.3011	8.7	10	11.2	12.9	14.2	16.4	19
02:05	29	13.0986	0.12665	-0.3022	8.8	10.1	11.4	13.1	14.4	16.7	19.3
02:06	30	13.2546	0.12675	-0.3033	9	10.3	11.6	13.3	14.7	17	19.6
02:07	31	13.4556	0.12685	-0.3055	9.1	10.4	11.7	13.5	14.9	17.3	19.9
02:08	32	13.649	0.12695	-0.3059	9.2	10.6	11.9	13.7	15.1	17.5	20.1
02:09	33	13.8962	0.12725	-0.3065	9.3	10.8	12.1	13.9	15.4	17.7	20.4
02:10	34	14.0855	0.12789	-0.3075	9.4	10.9	12.3	14.1	15.7	17.9	20.7
02:11	35	14.3656	0.12895	-0.3085	9.6	11.1	12.5	14.4	15.9	18.1	20.9
03:00	36	14.5236	0.12899	-0.3089	9.7	11.2	12.7	14.6	16.1	18.4	21.2
03:01	37	14.7563	0.12912	-0.3099	9.8	11.4	12.9	14.8	16.4	18.7	21.5
03:02	38	14.9853	0.12955	-0.3101	9.9	11.6	13.1	15	16.7	19	21.9
03:03	39	15.1526	0.12965	-0.3105	10.1	11.7	13.2	15.2	16.9	19.3	22.3

03:04	40	15.3255	0.12985	-0.3109	10.2	11.8	13.4	15.4	17.2	19.5	22.6
03:05	41	15.5256	0.12999	-0.3151	10.3	12	13.6	15.6	17.5	19.9	22.9
03:06	42	15.79	0.13001	-0.3155	10.4	12.1	13.8	15.8	17.6	20.1	23.3
03:07	43	15.9936	0.13058	-0.3165	10.5	12.2	14	16	17.9	20.4	23.6
03:08	44	16.1553	0.13099	-0.3171	10.6	12.3	14.2	16.2	18.1	20.7	24
03:09	45	16.3256	0.13121	-0.3181	10.7	12.5	14.4	16.4	18.4	20.9	24.4
03:10	46	16.5633	0.13125	-0.3189	10.8	12.7	14.6	16.6	18.7	21.2	24.8
03:11	47	16.7897	0.13136	-0.3191	10.9	12.8	14.8	16.8	19	21.5	25.2
04:00	48	16.8964	0.13145	-0.3199	11	12.9	14.9	16.9	19.2	21.7	25.5
04:01	49	17.0923	0.13199	-0.3201	11.1	13	15	17.1	19.4	22	25.8
04:02	50	17.1236	0.13201	-0.3209	11.2	13.1	15.2	17.2	19.6	22.4	26.1
04:03	51	17.2357	0.13222	-0.3222	11.3	13.2	15.3	17.3	19.9	22.7	26.5
04:04	52	17.4567	0.13325	-0.3245	11.4	13.3	15.4	17.5	20.2	23.1	26.8
04:05	53	17.6899	0.13465	-0.3255	11.5	13.4	15.5	17.7	20.4	23.5	27
04:06	54	17.9364	0.13569	-0.3265	11.6	13.5	15.6	17.9	20.6	23.9	27.4
04:07	55	18.0235	0.13698	-0.3275	11.7	13.6	15.8	18	20.8	24.3	27.9
04:08	56	18.1255	0.13789	-0.3285	11.8	13.7	15.9	18.2	21	24.7	28.4
04:09	57	18.3257	0.13896	-0.3291	11.9	13.8	16.1	18.4	21.2	25	28.9
04:10	58	18.5212	0.13963	-0.3321	12	13.9	16.2	18.6	21.4	25.3	29.3
04:11	59	18.6555	0.13998	-0.3329	12.1	14	16.3	18.7	21.7	25.7	29.6
05:00	60	18.7532	0.14005	-0.3333	12.2	14.1	16.4	18.8	21.9	26	30.1

Table (3): shows Egyptian L, M and S parameters and Z score length/height for age for boys from birth to 5 years

Length/Height-for-age BOYS<br>Egyptian Z-score birth to 5 Years												
Y:M	M	Mean	S	L	-3SD	-2SD	-1SD	Median	1SD	2SD	3SD
00:00	0	49.153	2.03561	1	43.5	45.3	47.3	49.2	51.4	53.1	55.1
00:01	1	53.9523	2.12788	1	48	50	52	54	56.1	58	60.1
00:02	2	57.6523	2.20612	1	51.6	53.7	55.7	57.7	60	62	63.9
00:03	3	60.7693	2.28023	1	54.5	56.5	58.6	60.8	63.1	64.9	67.2
00:04	4	63.1552	2.25325	1	56.8	59	61.1	63.2	65.4	67.6	69.5
00:05	5	65.2588	2.30724	1	58.9	60.8	63.1	65.3	67.5	69.6	71.8
00:06	6	66.7996	2.37619	1	60.4	62.4	64.8	66.8	69.4	71.4	73.5
00:07	7	68.4523	2.36431	1	62	64.2	66.2	68.4	70.9	73.1	75.2
00:08	8	59.8632	2.41595	1	63.1	65.4	67.7	69.9	72.3	74.5	76.8
00:09	9	71.1125	2.44701	1	64.5	66.8	69	71.2	73.7	76.1	78.2
00:10	10	72.425	2.46467	1	65.6	67.9	70.2	72.5	75.1	77.4	79.6
00:11	11	73.8269	2.53705	1	66.9	69	71.5	73.9	76.5	78.8	81.1
01:00	12	74.9632	2.43733	1	67.8	70.2	72.6	75.1	77.5	79.9	82.5
01:01	13	76.2589	2.54015	1	69	71.2	73.7	76.3	78.8	81.2	83.7
01:02	14	77.2456	2.72754	1	69.6	72.3	74.8	77.3	80.1	82.5	85.1
01:03	15	78.4563	2.77507	1	70.7	73.2	75.9	78.5	81.2	83.8	86.2
01:04	16	79.5686	2.82069	1	71.6	74.2	76.9	79.5	82.4	85	87.4
01:05	17	80.5025	2.85609	1	72.5	75.1	77.8	80.6	83.4	85.9	88.7
01:06	18	81.503	2.95455	1	73.5	76.1	78.8	81.6	84.6	87.2	90
01:07	19	82.5621	2.8604	1	74.2	77	79.8	82.6	85.5	88.3	91.1
01:08	20	83.426	3.01813	1	75.1	77.8	80.5	83.5	86.6	89.3	92.2
01:09	21	84.4863	2.97262	1	75.8	78.5	81.4	84.5	87.5	90.3	93.3
01:10	22	85.2665	2.99591	1	76.4	79.5	82.3	85.3	88.6	91.4	94.5
01:11	23	86.1596	3.14902	1	77.3	80.3	83.2	86.2	89.4	92.5	95.4
02:00	24	86.925	3.29912	1	77.6	80.6	83.8	87	90.3	93.3	96.3
02:01	25	87.2595	3.26118	1	78.1	81.1	84.2	87.3	90.7	93.9	96.9
02:02	26	87.9561	3.22325	1	78.6	81.9	84.9	88	91.6	94.7	97.8
02:03	27	88.9632	3.25771	1	79	82.3	85.6	88.9	92.4	95.7	98.9
02:04	28	89.7052	3.29217	1	79.7	83.1	86.4	89.8	93.3	96.5	99.8
02:05	29	90.5632	3.33189	1	80.2	83.7	87	90.6	94.1	97.3	100.7
02:06	30	91.152	3.37161	1	81	84.4	87.7	91.2	94.8	98.2	101.5
02:07	31	92.0359	3.45476	1	81.5	84.9	88.4	92.1	95.6	99	102.5
02:08	32	92.6078	3.53792	1	82.1	85.7	89	92.7	96.4	99.9	103.5
02:09	33	93.4256	3.59043	1	82.6	86.1	89.7	93.4	97.2	100.6	104.3
02:10	34	93.8962	3.64295	1	83.1	86.8	90.2	94	98	101.5	105
02:11	35	94.6302	3.70098	1	83.7	87.3	91	94.7	98.6	102.1	105.9
03:00	36	95.3215	3.75902	1	84.2	88	91.5	95.4	99.4	103	106.6
03:01	37	96.0258	3.78099	1	84.8	88.4	92.2	96.1	100.1	103.8	107.5
03:02	38	96.7035	3.80297	1	85.2	88.9	92.7	96.8	100.8	104.5	108.2
03:03	39	97.3753	3.82753	1	85.8	89.5	93.5	97.3	101.3	105.1	109

03:04	40	97.963	3.85209	1	86.2	90	94	98	102.1	105.9	109.7
03:05	41	98.4255	3.95937	1	86.8	90.5	94.5	98.5	102.7	106.8	110.5
03:06	42	99.1475	4.06665	1	87.2	91.2	95	99.2	103.3	107.4	111.1
03:07	43	99.8255	4.08487	1	87.5	91.5	95.5	99.8	104.1	108	112
03:08	44	100.302	4.10309	1	88	92.3	96.2	100.4	104.6	108.9	112.8
03:09	45	100.836	4.07108	1	88.6	92.7	96.6	100.9	105.1	109.3	113.4
03:10	46	101.523	4.03906	1	89.1	93.2	97.3	101.6	105.8	110	114
03:11	47	102.035	4.13047	1	89.5	93.7	97.7	102.1	106.3	110.6	114.7
04:00	48	102.658	4.22135	1	90	94	98.3	102.7	107	111.3	115.3
04:01	49	103.126	4.28964	1	90.4	94.6	99	103.2	107.5	111.9	116
04:02	50	103.62	4.35793	1	90.9	95.2	99.4	103.7	108.3	112.6	116.9
04:03	51	104.523	4.4028	1	91.3	95.6	100	104.5	108.8	113.1	117.4
04:04	52	104.963	4.44767	1	91.8	96.2	100.4	105	109.4	113.8	118
04:05	53	105.385	4.47049	1	92.2	96.6	100.8	105.4	110.1	114.4	118.7
04:06	54	106.085	4.49331	1	92.7	97.2	101.4	106.1	110.6	115.1	119.3
04:07	55	106.485	4.43254	1	93.2	97.5	102	106.5	111.3	115.6	120
04:08	56	107.013	4.37177	1	93.4	98.1	102.4	107.1	111.8	116.3	120.7
04:09	57	107.533	4.47826	1	94	98.5	103	107.6	112.4	117	121.3
04:10	58	108.116	4.58475	1	94.5	99	103.6	108.2	112.9	117.6	122.1
04:11	59	108.678	4.73317	1	94.8	99.4	104	108.7	113.5	118.1	122.7
05:00	60	109.352	4.88159	1	95.3	100	104.6	109.4	114	118.6	123.5

Table (4): shows Egyptian L, M and S parameters and Z score length/height for age for girls from birth to 5 years.

Length/Height-for-age GIRLS<br>Egyptian Z-score Birth to 5 Years											
Y:M	M	Mean	S	L	-3SD	-2SD	-1SD	Median	1SD	2SD	3SD
00:00	0	48.7088	1.95506	1	42.9	44.6	46.6	48.8	50.5	52.4	54.1
00:01	1	52.9752	2.02092	1	47	49	51	53	55	57	59
00:02	2	56.4225	2.131	1	50.1	52.1	54.2	56.5	58.5	60.7	62.8
00:03	3	59.1558	2.25371	1	52.5	54.7	56.8	59.2	61.4	63.5	65.6
00:04	4	51.5236	2.33775	1	54.7	57	59	61.6	63.9	66	68
00:05	5	63.6528	2.39822	1	56.6	58.7	61	63.7	66	68	70.2
00:06	6	65.2058	2.27542	1	58	60.4	62.7	65.3	67.5	69.9	72
00:07	7	66.695	2.31323	1	59.4	62	64.2	66.8	69	71.4	73.8
00:08	8	68.1258	2.34244	1	60.8	63.2	65.6	68.2	70.5	73	75.4
00:09	9	69.5025	2.50832	1	62	64.5	67	69.6	72	74.6	77
00:10	10	70.9956	2.58213	1	63.3	65.7	68.2	71	73.3	76	78.4
00:11	11	72.0147	2.69632	1	64.5	67	69.5	72.1	74.8	77.2	79.9
01:00	12	73.321	2.78059	1	65.5	68	70.6	73.4	76	78.6	81.2
01:01	13	74.4562	2.83125	1	66.5	69.2	71.9	74.5	77.2	80	82.6
01:02	14	75.589	2.65588	1	67.6	70.3	73	75.6	78.5	81.2	84
01:03	15	76.6874	2.78145	1	68.5	71.1	74	76.7	79.7	82.5	85.2
01:04	16	77.8026	3.06008	1	69.5	72.1	75	77.8	81	83.8	86.5
01:05	17	78.7962	3.11168	1	70.2	73.1	76.1	78.8	82	85	87.8
01:06	18	79.6147	3.16055	1	71.1	74	77.1	79.7	83	86	89
01:07	19	80.6652	3.33146	1	72	75	77.9	80.7	84.2	87	90
01:08	20	81.6544	3.3281	1	73	76	79	81.7	85.1	88.2	91.2
01:09	21	82.6201	3.14316	1	73.7	76.8	79.8	82.7	86.3	89.4	92.5
01:10	22	83.6735	3.33289	1	74.5	77.6	80.6	83.6	87.2	90.5	93.5
01:11	23	84.5001	3.3841	1	75.3	78.4	81.5	84.6	88.2	91.5	94.6
02:00	24	85.3046	3.57202	1	75.9	79	82.2	85.4	89.1	92.4	95.5
02:01	25	85.9075	3.56202	1	76.3	79.5	82.7	86	89.6	93	96.3
02:02	26	96.5174	3.58127	1	76.9	80.1	83.4	86.6	90.4	93.8	97
02:03	27	97.2581	3.56202	1	77.5	80.7	84	87.3	91.3	94.5	98
02:04	28	88.2741	3.52696	1	78	81.3	85	88.3	92	95.5	98.9
02:05	29	89.1234	3.51258	1	78.6	82	85.6	89.2	93	96.4	99.8
02:06	30	89.8236	3.52521	1	79.3	82.8	86.4	89.9	93.8	97.2	100.8
02:07	31	90.5721	3.72477	1	80	83.5	87	90.6	94.5	98	101.8
02:08	32	91.3753	3.73477	1	80.5	84	87.9	91.4	95.3	99	102.7
02:09	33	92.135	3.83698	1	81	84.9	88.5	92.2	96	99.9	103.5
02:10	34	92.8301	4.1002	1	81.6	85.5	89	92.9	97	100.7	104.3
02:11	35	93.6852	3.6875	1	82.3	86	89.7	93.7	97.6	101.2	105
03:00	36	94.3756	3.77889	1	82.9	86.6	90.3	94.6	98.3	102.1	106
03:01	37	94.9334	3.87652	1	83.3	87.2	91	95	99	103	106.9
03:02	38	95.5778	4.02487	1	84	87.8	91.6	95.6	99.8	103.8	107.6
03:03	39	96.3651	4.02543	1	84.5	88.5	92.3	96.4	100.6	104.5	108.5

03:04	40	97.1132	4.06543	1	85	89	93	97.2	101.3	105.1	109.2
03:05	41	97.7258	4.07524	1	85.4	89.6	93.6	97.7	102	106	110
03:06	42	98.3358	4.08252	1	86	90	94.2	98.3	102.6	106.7	110.7
03:07	43	98.9352	4.07258	1	86.6	90.6	94.9	99	103.3	107.3	111.5
03:08	44	99.5321	4.0639	1	87	91.3	95.5	99.6	104	108	112.2
03:09	45	100.114	4.12584	1	87.5	91.8	96	100.2	104.6	108.9	113
03:10	46	100.903	4.25164	1	88	92.4	96.5	101	105.2	109.5	113.8
03:11	47	101.476	4.25875	1	88.5	92.9	97	101.5	106	110.2	114.5
04:00	48	101.82	4.28939	1	89	93.4	97.6	101.9	106.6	110.8	115.3
04:01	49	102.583	4.25875	1	89.5	93.9	98.2	102.5	107.1	111.5	116
04:02	50	103.145	4.32136	1	90	94.2	98.6	103.2	107.8	112.1	116.7
04:03	51	103.926	4.58752	1	90.4	94.7	99.3	103.9	108.5	112.9	117.3
04:04	52	104.218	4.65084	1	91	95.4	99.7	104.3	109	113.6	118
04:05	53	105.926	4.58725	1	91.3	95.8	100.4	105	109.7	114	118.6
04:06	54	105.403	4.76789	1	91.7	96.2	100.9	105.5	110.2	114.8	119.2
04:07	55	106.002	4.75987	1	92.1	96.8	101.5	106	110.7	115.5	120
04:08	56	106.404	4.7061	1	92.6	97.2	102	106.5	111.5	116	120.5
04:09	57	106.983	4.5875	1	93	97.6	102.4	107	112	116.5	121.3
04:10	58	107.503	4.6589	1	93.5	98.2	103	107.6	112.6	117.2	122
04:11	59	108.158	4.68754	1	94	98.7	103.5	108.1	113	117.9	122.6
05:00	60	108.685	4.65815	1	94.3	99	104	108.7	113.7	118.5	123.2

Table (5): shows Egyptian L, M and S parameters and Z score Wight for length/height for boys from birth to 5 years

Weight-for-length/height BOYS Egyptian Z-score Birth to 5 Years										
Length/height	Mean	S	L	-3SD	-2SD	-1SD	Median	1SD	2SD	3SD
45	2.4598	0.09116	-0.3531	1.8	2	2.3	2.5	2.8	3.1	3.4
45.5	2.5236	0.09119	-0.3531	1.9	2.1	2.4	2.6	2.9	3.2	3.5
46	2.6985	0.09120	-0.3531	2	2.2	2.5	2.7	2.9	3.3	3.6
46.5	2.7152	0.09122	-0.3531	2	2.3	2.6	2.7	3	3.3	3.7
47	2.7568	0.09123	-0.3531	2.1	2.3	2.7	2.8	3.1	3.4	3.8
47.5	2.8245	0.09125	-0.3531	2.2	2.4	2.7	2.9	3.2	3.5	3.9
48	2.9354	0.09126	-0.3531	2.3	2.5	2.8	3	3.3	3.7	4
48.5	3.0214	0.09128	-0.3531	2.3	2.6	2.9	3.1	3.4	3.8	4.1
49	3.1754	0.09132	-0.3531	2.4	2.7	3	3.2	3.5	3.9	4.3
49.5	3.215	0.09134	-0.3531	2.5	2.7	3.1	3.3	3.6	4	4.4
50	3.3012	0.09136	-0.3531	2.6	2.8	3.1	3.4	3.8	4.1	4.5
50.5	3.4752	0.09137	-0.3531	2.7	2.9	3.2	3.5	3.9	4.2	4.7
51	3.5752	0.09139	-0.3531	2.8	3	3.3	3.7	4	4.4	4.8
51.5	3.6896	0.09140	-0.3531	2.9	3.1	3.4	3.8	4.2	4.5	4.9
52	3.8045	0.09141	-0.3531	3	3.2	3.6	3.9	4.3	4.6	5.1
52.5	3.9125	0.09142	-0.3531	3.1	3.3	3.7	4	4.4	4.7	5.2
53	4.0752	0.09144	-0.3531	3.2	3.4	3.8	4.1	4.6	4.9	5.4
53.5	4.1253	0.09146	-0.3531	3.3	3.5	3.9	4.3	4.7	5	5.6
54	4.3582	0.09147	-0.3531	3.4	3.6	4.1	4.4	4.8	5.2	5.7
54.5	4.4365	0.09149	-0.3531	3.5	3.8	4.3	4.6	5.1	5.3	5.9
55	4.6583	0.09151	-0.3531	3.6	3.9	4.4	4.7	5.2	5.5	6
55.5	4.8253	0.09152	-0.3531	3.7	4	4.5	4.8	5.3	5.6	6.2
56	4.9872	0.09154	-0.3531	3.9	4.1	4.6	4.9	5.5	5.7	6.3
56.5	5.0985	0.09155	-0.3531	4	4.2	4.7	5.1	5.6	5.9	6.5
57	5.2015	0.09157	-0.3531	4.1	4.4	4.8	5.2	5.7	6	6.7
57.5	5.4687	0.09159	-0.3531	4.2	4.5	4.9	5.4	5.9	6.2	6.9
58	5.6742	0.09160	-0.3531	4.3	4.6	5	5.6	6	6.3	7.1
58.5	5.7654	0.09162	-0.3531	4.5	4.8	5.2	5.7	6.2	6.4	7.4
59	5.9823	0.09163	-0.3531	4.6	4.9	5.3	5.9	6.3	6.6	7.5
59.5	6.0153	0.09165	-0.3531	4.7	5	5.4	6	6.5	6.8	7.7
60	6.2154	0.09167	-0.3531	4.8	5.1	5.5	6.2	6.7	7	7.9
60.5	6.3853	0.09168	-0.3531	4.9	5.3	5.7	6.3	6.8	7.2	8
61	6.4685	0.09170	-0.3531	5	5.4	5.8	6.4	6.9	7.3	8.1
61.5	6.6065	0.09172	-0.3531	5.1	5.5	5.9	6.6	7.1	7.5	8.3
62	6.7256	0.09173	-0.3531	5.2	5.6	6	6.7	7.2	7.6	8.5
62.5	6.8632	0.09175	-0.3531	5.3	5.7	6.2	6.8	7.4	7.7	8.6
63	8.925	0.09176	-0.3531	5.4	5.8	6.3	6.9	7.5	7.9	8.8
63.5	7.0523	0.09179	-0.3531	5.5	5.9	6.4	7	7.7	8.1	8.9
64	7.1263	0.09181	-0.3531	5.6	6	6.5	7.1	7.8	8.2	9
64.5	7.356	0.09182	-0.3531	5.7	6.1	6.6	7.3	7.9	8.3	9.2

65	7.4623	0.09183	-0.3531	5.8	6.2	6.7	7.4	8	8.5	9.4
65.5	7.5375	0.09184	-0.3531	5.9	6.4	6.8	7.5	8.1	8.6	9.5
67	7.6325	0.09186	-0.3531	6	6.5	6.9	7.6	8.3	8.7	9.7
67.5	7.8362	0.09187	-0.3531	6.1	6.6	7	7.8	8.4	8.9	9.8
68	7.9236	0.09188	-0.3531	6.2	6.7	7.2	7.9	8.5	9	10
68.5	8.0253	0.09189	-0.3531	6.3	6.8	7.3	8	8.7	9.1	10.2
69	8.1263	0.09190	-0.3531	6.4	6.9	7.4	8.1	8.8	9.3	10.3
69.5	8.2336	0.09191	-0.3531	6.5	6.9	7.5	8.2	8.9	9.5	10.5
70	8.30125	0.09192	-0.3531	6.6	7	7.6	8.2	9	9.6	10.6
70.5	8.3923	0.09194	-0.3531	6.7	7.1	7.7	8.3	9.1	9.8	10.7
71	8.4523	0.09196	-0.3531	6.8	7.2	7.8	8.4	9.3	10	10.9
71.5	8.5315	0.09198	-0.3531	6.9	7.3	7.9	8.5	9.4	10.1	11
72	8.6359	0.09199	-0.3531	7	7.4	8	8.6	9.5	10.2	11.1
72.5	8.7251	0.09201	-0.3531	7	7.5	8.1	8.7	9.6	10.3	11.2
73	8.832	0.09203	-0.3531	7.1	7.6	8.2	8.8	9.7	10.5	11.4
73.5	8.90125	0.09204	-0.3531	7.2	7.8	8.2	8.9	9.9	10.6	11.5
74	9.09856	0.09205	-0.3531	7.3	7.9	8.3	9.1	10	10.7	11.6
74.5	9.2563	0.09207	-0.3531	7.4	7.9	8.4	9.2	10.2	10.9	11.7
75	9.3785	0.09208	-0.3531	7.5	8	8.5	9.3	10.3	11	11.9
75.5	9.462	0.09210	-0.3531	7.6	8.1	8.6	9.4	10.4	11.1	12
76	9.5145	0.09211	-0.3531	7.7	8.2	8.7	9.5	10.5	11.2	12.2
76.5	9.6325	0.09213	-0.3531	7.8	8.3	8.8	9.6	10.7	11.3	12.3
77	9.7531	0.09214	-0.3531	7.9	8.4	8.9	9.7	10.8	11.5	12.5
77.5	9.80086	0.09215	-0.3531	7.9	8.5	8.9	9.8	10.9	11.6	12.6
78	9.8891	0.09216	-0.3531	8	8.5	9	9.8	11	11.7	12.8
78.5	9.90125	0.09218	-0.3531	8.1	8.6	9.1	9.9	11.1	11.8	12.9
79	10.0126	0.09219	-0.3531	8.2	8.7	9.2	10	11.2	12	13.1
79.5	10.1257	0.09220	-0.3531	8.2	8.8	9.3	10.1	11.3	12.1	13.2
80	10.2752	0.09222	-0.3531	8.3	8.9	9.4	10.2	11.4	12.2	13.3
80.5	10.3856	0.09223	-0.3531	8.4	8.9	9.5	10.3	11.5	12.3	13.5
81	10.4253	0.09224	-0.3531	8.5	9	9.6	10.4	11.6	12.4	13.6
81.5	10.5147	0.09226	-0.3531	8.6	9	9.7	10.5	11.7	12.5	13.7
82	10.6385	0.09228	-0.3531	8.7	9.1	9.8	10.6	11.8	12.6	13.8
82.5	10.7863	0.09229	-0.3531	8.7	9.2	9.9	10.7	12	12.8	14
83	10.8256	0.09230	-0.3531	8.8	9.3	10	10.8	12.1	12.9	14.1
83.5	10.9362	0.09231	-0.3531	8.9	9.4	10.1	10.9	12.2	13	14.2
84	11.0532	0.09233	-0.3531	9	9.5	10.2	11	12.3	13.1	14.4
84.5	11.1925	0.09235	-0.3531	9.1	9.6	10.3	11.2	12.5	13.2	14.5
85	11.2635	0.09236	-0.3531	9.2	9.7	10.4	11.3	12.6	13.3	14.7
85.5	11.4752	0.09237	-0.3531	9.3	9.8	10.5	11.4	12.8	13.4	14.8
86	11.5356	0.09239	-0.3531	9.4	9.9	10.6	11.5	12.9	13.6	15
86.5	11.6856	0.09240	-0.3531	9.5	10	10.8	11.6	13.1	13.7	15.2
87	11.8253	0.09241	-0.3531	9.6	10.1	10.9	11.8	13.2	13.8	15.3
87.5	11.9027	0.09242	-0.3531	9.7	10.2	11	11.9	13.3	14	15.5
88	12.0823	0.09243	-0.3531	9.8	10.3	11.1	12	13.4	14.1	15.6
88.5	12.1526	0.09247	-0.3531	9.8	10.5	11.2	12.1	13.5	14.3	15.8

89	12.2581	0.09249	-0.3531	9.9	10.6	11.3	12.2	13.6	14.4	15.9
89.5	12.3635	0.09250	-0.3531	10	10.7	11.4	12.3	13.8	14.5	16
90	12.59675	0.09251	-0.3531	10.1	10.8	11.5	12.5	14	14.6	16.2
90.5	12.63589	0.09253	-0.3531	10.2	10.9	11.7	12.6	14.1	14.7	16.3
91	12.7452	0.09254	-0.3531	10.3	11	11.8	12.7	14.2	15.9	16.5
91.5	12.8102	0.09255	-0.3531	10.4	11.1	11.9	12.8	14.3	15	16.6
92	12.9898	0.09256	-0.3531	10.5	11.2	12	13	14.5	15.1	16.8
92.5	13.1754	0.09258	-0.3531	10.6	11.3	12.1	13.1	14.6	15.3	16.9
93	13.2586	0.09259	-0.3531	10.7	11.4	12.2	13.2	14.7	15.4	17.1
93.5	13.3254	0.09260	-0.3531	10.8	11.5	12.3	13.3	14.9	15.5	17.2
94	13.4789	0.09253	-0.3531	10.9	11.6	12.4	13.4	15	15.7	17.3
94.5	13.6102	0.09251	-0.3531	11	11.7	12.5	13.6	15.1	15.8	17.4
95	13.7452	0.09252	-0.3531	11.1	11.8	12.6	13.7	15.3	15.9	17.6
95.5	13.8751	0.09242	-0.3531	11.2	11.9	12.7	13.8	15.4	16.1	17.7
96	13.9953	0.09243	-0.3531	11.3	12	12.9	13.9	15.5	16.2	17.9
96.5	14.0852	0.09241	-0.3531	11.4	12.1	13	14	15.7	16.4	18.1
97	14.2102	0.09232	-0.3531	11.5	12.2	13.1	14.2	15.8	16.5	18.2
97.5	14.3451	0.09231	-0.3531	11.6	12.3	13.2	14.3	16	16.7	18.3
98	14.4617	0.09230	-0.3531	11.7	12.4	13.3	14.4	16.1	16.8	18.5
98.5	14.583	0.09227	-0.3531	11.8	12.5	13.4	14.5	16.2	17	18.6
99	14.6246	0.09226	-0.3531	11.9	12.6	13.5	14.6	16.4	17.1	18.8
99.5	14.7035	0.092223	-0.3531	12	12.8	13.7	14.7	16.5	17.3	19
100	14.8995	0.092221	-0.3531	12.2	12.9	13.8	14.9	16.6	17.5	19.1
100.5	15.0751	0.09215	-0.3531	12.3	13	13.9	15	16.7	17.6	19.2
101	15.1211	0.09213	-0.3531	12.4	13.1	14	15.1	16.8	17.8	19.4
101.5	15.3254	0.09211	-0.3531	12.5	13.2	14.1	15.3	17	18	19.6
102	15.4114	0.09208	-0.3531	12.6	13.3	14.3	15.4	17.1	18.2	19.8
102.5	15.5985	0.09205	-0.3531	12.7	13.4	14.4	15.6	17.2	18.3	20
103	15.7585	0.09203	-0.3531	12.8	13.6	14.5	15.7	17.4	18.4	20.2
103.5	15.9201	0.09201	-0.3531	12.9	13.7	14.7	15.9	17.5	18.6	20.4
104	16.1545	0.09198	-0.3531	13	13.8	14.8	16.1	17.6	18.8	20.6
104.5	16.3414	0.09197	-0.3531	13.1	13.9	14.9	16.3	17.8	19	20.8
105	16.4252	0.09196	-0.3531	13.3	14	15	16.4	18	19.1	21.1
105.5	16.6125	0.09195	-0.3531	13.4	14.2	15.2	16.6	18.1	19.3	21.3
106	16.7775	0.09193	-0.3531	13.5	14.3	15.3	16.7	18.3	19.5	21.5
106.5	16.9888	0.09192	-0.3531	13.6	14.4	15.4	16.9	18.4	19.7	21.8
107	17.0221	0.0919	-0.3531	13.7	14.5	15.6	17	18.5	19.9	22
107.5	17.23322	0.09189	-0.3531	13.8	14.6	15.7	17.2	18.6	20.1	22.3
108	17.3333	0.09187	-0.3531	13.9	14.7	15.9	17.3	18.7	20.3	22.5
108.5	17.4525	0.09186	-0.3531	14	14.9	16	17.4	18.9	20.5	22.8
109	17.6325	0.09184	-0.3531	14.2	15	16.2	17.6	19.1	20.7	23.1
109.5	17.7852	0.09182	-0.3531	14.3	15.1	16.3	17.8	19.3	20.9	23.4
110	17.90258	0.09181	-0.3531	14.4	15.3	16.5	17.9	19.5	21.1	23.7
110.5	18.0121	0.09178	-0.3531	14.5	15.4	16.6	18.1	19.7	21.3	24
111	18.3001	0.09176	-0.3531	14.6	15.6	16.8	18.3	19.9	21.6	24.2
111.5	18.5236	0.09175	-0.3531	14.8	15.7	17	18.5	20.1	21.8	24.4

112	18.75221	0.09173	-0.3531	14.9	15.9	17.1	18.7	20.3	22	24.7
112.5	18.9363	0.09169	-0.3531	15	16	17.3	18.9	20.5	22.2	25
113	19.0124	0.09167	-0.3531	15.1	16.1	17.5	19.1	20.8	22.5	25.2
113.5	19.2301	0.09165	-0.3531	15.3	16.3	17.6	19.3	21	22.7	25.5
114	19.4257	0.09164	-0.3531	15.4	16.4	17.8	19.5	21.2	22.9	25.8
114.5	19.6125	0.09162	-0.3531	15.5	16.6	18	19.7	21.4	23.2	26
115	19.9856	0.09161	-0.3531	15.7	16.7	18.1	19.9	21.6	23.4	26.3
115.5	20.0125	0.09159	-0.3531	15.8	16.9	18.3	20.1	21.9	23.6	26.6
116	20.2012	0.09157	-0.3531	15.9	17	18.5	20.3	22.1	23.9	26.9
116.5	20.4125	0.09156	-0.3531	16.1	17.2	18.6	20.5	22.4	24.2	27.2
117	20.7544	0.09154	-0.3531	16.1	17.3	19	20.8	22.8	24.4	27.5
117.5	21.0325	0.09153	-0.3531	16.3	17.5	19.2	21	23	24.6	27.8
118	21.2523	0.09151	-0.3531	16.4	17.6	19.4	21.2	23.2	24.9	28
118.5	21.3579	0.0915	-0.3531	16.5	17.8	19.5	21.4	23.5	25.1	28.3
119	21.568	0.09148	-03531	16.7	17.9	19.7	21.6	23.7	25.4	28.6
119.5	21.7452	0.09147	-0.3531	16.8	18.1	19.9	21.8	23.9	25.6	28.9
120	21.9889	0.09143	-0.3531	16.9	18.3	20.1	22	24.2	25.9	29.2

Table (6): shows Egyptian L, M and S parameters and Z score Wight for length/height for girls from birth to 5 years

Weight-for- Length/height GIRLS Egyptian Z-score Birth to 5 Years										
Length/height	Mean	S	L	-3SD	-2SD	-1SD	Median	1SD	2SD	3SD
45	2.6632	0.09018	-0.3732	2	2.1	2.4	2.6	2.8	3.1	3.4
45.5	2.7258	0.09024	-0.3732	2	2.2	2.5	2.7	2.9	3.2	3.5
46	2.8265	0.09029	-0.3732	2.1	2.3	2.6	2.8	3	3.3	3.6
46.5	2.8856	0.09034	-0.3732	2.2	2.4	2.7	2.8	3.1	3.4	3.8
47	2.9158	0.09036	-0.3732	2.2	2.4	2.7	2.9	3.2	3.5	3.9
47.5	2.9985	0.09038	-0.3732	2.3	2.5	2.8	3	3.3	3.6	4
48	3.025	0.09039	-0.3732	2.3	2.6	2.8	3.1	3.6	3.8	4.1
48.5	3.1526	0.09042	-0.3732	2.4	2.7	2.9	3.2	3.7	3.9	4.3
49	3.2986	0.09045	-0.3732	2.4	2.8	3	3.3	3.8	4	4.5
49.5	3.4266	0.09048	-0.3732	2.5	2.9	3.1	3.4	3.9	4.1	4.7
50	3.5689	0.09051	-0.3732	2.5	3	3.2	3.5	4	4.2	4.8
50.5	3.6589	0.09056	-0.3732	2.6	3.1	3.3	3.6	4.1	4.3	5
51	3.7852	0.09059	-0.3732	2.7	3.2	3.5	3.7	4.2	4.4	5.2
51.5	3.9052	0.09063	-0.3732	2.7	3.3	3.6	3.9	4.3	4.5	5.3
52	4.0256	0.09065	-0.3732	2.8	3.4	3.7	4	4.5	4.7	5.5
52.5	4.1256	0.09067	-0.3732	2.9	3.5	3.8	4.1	4.6	4.8	5.7
53	4.2563	0.09069	-0.3732	3	3.6	3.9	4.2	4.7	4.9	5.9
53.5	4.3985	0.09070	-0.3732	3.1	3.7	4	4.3	4.9	5	6
54	4.4682	0.09072	-0.3732	3.2	3.8	4.1	4.4	5	5.1	6.2
54.5	4.5986	0.09074	-0.3732	3.3	3.9	4.2	4.6	5.1	5.3	6.3
55	4.7582	0.09076	-0.3732	3.4	4	4.3	4.7	5.3	5.4	6.5
55.5	4.8632	0.09078	-0.3732	3.5	4.1	4.4	4.8	5.4	5.6	6.7
56	4.985	0.09081	-0.3732	3.6	4.2	4.5	4.9	5.6	5.7	6.9
56.5	5.0256	0.09082	-0.3732	3.7	4.3	4.6	5	5.7	5.8	7
57	5.1523	0.09085	-0.3732	3.8	4.4	4.8	5.1	5.8	6	7.1
57.5	5.2632	0.09086	-0.3732	3.9	4.5	4.9	5.2	6	6.1	7.3
58	5.3258	0.09087	-0.3732	4	4.6	5	5.3	6.1	6.2	7.5
58.5	5.4025	0.09088	-0.3732	4.1	4.7	5.1	5.4	6.3	6.4	7.6
59	5.5332	0.09089	-0.3732	4.2	4.8	5.2	5.5	6.4	6.5	7.7
59.5	5.6375	0.09091	-0.3732	4.3	4.9	5.3	5.6	6.5	6.6	7.9
60	5.7952	0.09093	-0.3732	4.5	5	5.4	5.7	6.7	6.8	8
60.5	5.8963	0.09095	-0.3732	4.6	5.1	5.5	5.8	6.8	7	8.2
61	5.9012	0.09095	-0.3732	4.7	5.2	5.6	5.9	7	7.2	8.3
61.5	6.0967	0.09097	-0.3732	4.8	5.3	5.7	6.1	7.1	7.4	8.5
62	6.2586	0.09098	-0.3732	4.9	5.4	5.8	6.2	7.2	7.6	8.6
62.5	6.3745	0.09099	-0.3732	5	5.5	5.9	6.3	7.4	7.7	8.8
63	6.5014	0.09108	-0.3732	5.1	5.6	6	6.5	7.5	7.9	9
63.5	6.7025	0.09109	-0.3732	5.2	5.7	6.1	6.7	7.7	8.1	9.2
64	6.8759	0.09112	-0.3732	5.2	5.8	6.2	6.8	7.8	8.3	9.4
64.5	6.9015	0.09116	-0.3732	5.3	5.9	6.3	6.9	8	8.4	9.6

65	7.0985	0.09118	-0.3732	5.4	6	6.5	7.1	8.1	8.6	9.8
65.5	7.1925	0.09119	-0.3732	5.5	6.1	6.6	7.2	8.2	8.7	9.9
67	7.3987	0.09120	-0.3732	5.6	6.2	6.8	7.4	8.4	8.9	10.2
67.5	7.5012	0.09124	-0.3732	5.7	6.3	6.9	7.5	8.5	9	10.5
68	7.6612	0.09126	-0.3732	5.8	6.4	7	7.6	8.6	9.1	10.6
68.5	7.7421	0.09127	-0.3732	5.9	6.5	7.1	7.7	8.8	9.2	10.8
69	7.824	0.09129	-0.3732	6	6.6	7.2	7.8	8.9	9.4	11
69.5	7.9124	0.09131	-0.3732	6.1	6.7	7.3	7.9	9	9.5	11.1
70	8.0245	0.09133	-0.3732	6.2	6.8	7.4	8	9.1	9.7	11.2
70.5	8.1753	0.09135	-0.3732	6.3	6.9	7.5	8.1	9.2	9.8	11.5
71	8.2345	0.09136	-0.3732	6.4	7.1	7.6	8.2	9.3	9.9	11.6
71.5	8.3752	0.09138	-0.3732	6.5	7.2	7.7	8.3	9.4	10.1	11.8
72	8.4963	0.09139	-0.3732	6.6	7.3	7.8	8.4	9.5	10.2	11.9
72.5	8.5012	0.09141	-0.3732	6.7	7.3	7.9	8.5	9.7	10.3	12
73	8.6725	0.09142	-0.3732	6.8	7.4	7.9	8.6	9.8	10.4	12.1
73.5	8.7012	0.09144	-0.3732	6.8	7.5	8	8.7	9.9	10.5	12.3
74	8.8858	0.09146	-0.3732	6.9	7.6	8.1	8.8	10	10.7	12.4
74.5	8.9145	0.09147	-0.3732	7	7.7	8.2	8.9	10.1	10.8	12.5
75	9.0125	0.09149	-0.3732	7.1	7.8	8.2	9	10.2	10.9	12.6
75.5	9.1563	0.09151	-0.3732	7.2	7.9	8.3	9.1	10.3	11	12.8
76	9.2236	0.09152	-0.3732	7.3	7.9	8.4	9.2	10.4	11.1	12.9
76.5	9.3521	0.09154	-0.3732	7.3	8	8.5	9.3	10.5	11.2	13.1
77	9.4865	0.09155	-0.3732	7.4	8.1	8.6	9.4	10.6	11.3	13.3
77.5	9.5175	0.09157	-0.3732	7.4	8.2	8.7	9.5	10.7	11.5	13.4
78	9.6073	0.09159	-0.3732	7.5	8.2	8.8	9.6	10.9	11.6	13.6
78.5	9.7892	0.09160	-0.3732	7.5	8.3	8.9	9.7	11	11.7	13.8
79	9.8034	0.09162	-0.3732	7.6	8.4	9	9.8	11.1	11.8	14
79.5	9.9723	0.09164	-0.3732	7.7	8.5	9.1	9.9	11.2	11.9	14.1
80	10.0145	0.09166	-0.3732	7.7	8.6	9.2	10	11.3	12	14.3
80.5	10.0793	0.09168	-0.3732	7.8	8.7	9.3	10	11.4	12.2	14.5
81	10.1523	0.09169	-0.3732	7.9	8.8	9.4	10.1	11.5	12.3	14.6
81.5	10.2352	0.09170	-0.3732	8	8.8	9.6	10.2	11.7	12.4	14.8
82	10.3521	0.09173	-0.3732	8.1	9	9.7	10.3	11.8	12.5	15
82.5	10.5412	0.09174	-0.3732	8.2	9.1	9.8	10.5	11.9	12.6	15.2
83	10.6782	0.09175	-0.3732	8.3	9.2	9.9	10.6	12	12.8	15.3
83.5	10.7028	0.09177	-0.3732	8.4	9.3	10	10.7	12.1	12.9	15.5
84	10.8345	0.09179	-0.3732	8.5	9.4	10.1	10.8	12.2	13	15.7
84.5	10.996	0.09181	-0.3732	8.6	9.5	10.2	10.9	12.3	13.2	15.9
85	11.0745	0.09182	-0.3732	8.7	9.6	10.3	11	12.4	13.3	16
85.5	11.1962	0.09183	-0.3732	8.7	9.7	10.4	11.2	12.5	13.4	16.1
86	11.352	0.09184	-0.3732	8.8	9.8	10.5	11.3	12.6	13.5	16.3
86.5	11.4852	0.09186	-0.3732	8.9	9.9	10.6	11.4	12.7	13.6	16.4
87	11.5634	0.09187	-0.3732	9	10	10.7	11.5	12.8	13.8	16.5
87.5	11.6504	0.09188	-0.3732	9.1	10.1	10.8	11.6	12.9	14	16.6
88	11.7152	0.09189	-0.3732	9.2	10.2	10.9	11.7	13	14.2	16.7
88.5	11.9325	0.09190	-0.3732	9.3	10.3	11	11.9	13.1	14.4	16.9

89	12.0153	0.09191	-0.3732	9.4	10.4	11.2	12	13.2	14.5	17
89.5	12.1523	0.09192	-0.3732	9.5	10.6	11.3	12.1	13.3	14.6	17.2
90	12.2156	0.09194	-0.3732	9.6	10.7	11.4	12.2	13.5	14.8	17.3
90.5	12.3985	0.09196	-0.3732	9.7	10.8	11.5	12.4	13.6	15	17.4
91	12.4562	0.09198	-0.3732	9.8	10.9	11.6	12.5	13.7	15.2	17.5
91.5	12.6523	0.09199	-0.3732	9.9	11	11.7	12.6	13.8	15.3	17.7
92	12.7852	0.09201	-0.3732	10	11.2	11.8	12.7	14	15.5	17.9
92.5	12.8014	0.09204	-0.3732	10.1	11.4	12	12.8	14.1	15.7	18.1
93	12.9985	0.09205	-0.3732	10.2	11.5	12.1	13	14.2	15.9	18.2
93.5	13.0452	0.09206	-0.3732	10.3	11.7	12.2	13.1	14.3	16.1	18.4
94	13.2571	0.09208	-0.3732	10.4	11.8	12.3	13.2	14.5	16.2	18.6
94.5	13.4125	0.09209	-0.3732	10.5	12	12.4	13.4	14.7	16.4	18.8
95	13.5252	0.09210	-0.3732	10.6	12.2	12.6	13.5	14.8	16.5	19
95.5	13.6752	0.09211	-0.3732	10.6	12.4	12.7	13.6	15	16.7	19.2
96	13.7301	0.09213	-0.3732	10.7	12.5	12.8	13.7	15.2	16.8	19.4
96.5	13.8963	0.09214	-0.3732	10.8	12.7	12.9	13.9	15.4	16.9	19.6
97	14.0152	0.09215	-0.3732	10.9	12.8	13	14	15.5	17.1	19.8
97.5	14.1752	0.09216	-0.3732	11	12.9	13.2	14.1	15.7	17.3	20
98	14.1963	0.09218	-0.3732	11.1	13	13.3	14.2	15.9	17.4	20.2
98.5	14.2986	0.09219	-0.3732	11.2	13.1	13.4	14.3	16	17.6	20.4
99	14.4563	0.09220	-0.3732	11.4	13.2	13.5	14.5	16.2	17.7	20.6
99.5	14.6588	0.09222	-0.3732	11.5	13.3	13.6	14.6	16.4	17.9	20.7
100	14.8752	0.09223	-0.3732	11.6	13.5	13.7	14.8	16.6	18	20.9
100.5	14.9852	0.09224	-0.3732	11.7	13.6	13.8	14.9	16.8	18.2	21.1
101	15.0123	0.09226	-0.3732	11.8	13.7	14	15	16.9	18.4	21.3
101.5	15.2221	0.09228	-0.3732	11.9	13.8	14.1	15.2	17.1	18.6	21.4
102	15.3752	0.09229	-0.3732	12	14	14.2	15.3	17.3	18.8	21.6
102.5	15.5145	0.09230	-0.3732	12.2	14.1	14.4	15.5	17.4	19	21.8
103	15.6321	0.09232	-0.3732	12.3	14.2	14.6	15.6	17.6	19.2	21.9
103.5	15.7526	0.09233	-0.3732	12.4	14.3	14.7	15.7	17.8	19.4	22.1
104	15.8632	0.09235	-0.3732	12.5	14.4	14.8	15.8	18	19.6	22.3
104.5	16.0256	0.09236	-0.3732	12.7	14.5	14.9	16	18.2	19.7	22.5
105	16.1756	0.09238	-0.3732	12.8	14.6	15.1	16.1	18.4	19.8	22.7
105.5	16.2015	0.09239	-0.3732	12.9	14.7	15.2	16.2	18.6	20	22.9
106	16.4021	0.09240	-0.3732	13	14.8	15.3	16.4	18.8	20.3	23.2
106.5	16.5412	0.09241	-0.3732	13.2	14.9	15.5	16.5	19	20.5	23.5
107	16.6123	0.09242	-0.3732	13.4	15	15.7	16.6	19.2	20.8	23.7
107.5	16.815	0.09243	-0.3732	13.5	15.1	15.8	16.8	19.4	21	24
108	16.9045	0.09248	-0.3732	13.7	15.3	15.9	16.9	19.6	21.2	24.3
108.5	17.0125	0.09249	-0.3732	13.8	15.5	16.1	17	19.8	21.4	24.6
109	17.2456	0.09250	-0.3732	13.9	15.6	16.2	17.2	20.1	21.6	24.8
109.5	17.4623	0.09251	-0.3732	14	15.7	16.4	17.5	20.4	21.9	25.1
110	17.7963	0.09253	-0.3732	14.2	15.9	16.5	17.7	20.6	22.2	25.4
110.5	17.9856	0.09254	-0.3732	14.3	16	16.7	17.9	20.8	22.5	25.7
111	18.0856	0.09255	-0.3732	14.4	16.4	16.8	18	21	22.8	26
111.5	18.1982	0.09256	-0.3732	14.6	16.5	17	18.2	21.3	23.1	26.3

112	18.3754	0.09258	-0.3732	14.7	16.7	17.2	18.4	21.5	23.4	26.6
112.5	18.5463	0.09259	-0.3732	14.8	16.9	17.4	18.6	21.8	23.7	26.8
113	18.7851	0.09260	-0.3732	15	17	17.6	18.8	22	23.9	27.1
113.5	19.0215	0.09253	-0.3732	15.2	17.2	17.8	19	22.3	24.2	27.4
114	19.2543	0.09251	-0.3732	15.3	17.4	18	19.2	22.5	24.4	27.8
114.5	19.4576	0.09252	-0.3732	15.4	17.5	18.2	19.4	22.8	24.7	28.1
115	19.6752	0.09242	-0.3732	15.6	17.6	18.4	19.6	23	24.9	28.4
115.5	19.8012	0.09243	-0.3732	15.7	17.8	18.6	19.8	23.3	25.2	28.7
116	20.0751	0.09241	-0.3732	15.9	17.9	18.8	20	23.6	25.5	29
116.5	20.1987	0.09232	-0.3732	16	18	19	20.2	23.8	25.7	29.3
117	20.4035	0.09231	-0.3732	16.1	18.1	19.2	20.4	24	26.1	29.6
117.5	20.6856	0.09230	-0.3732	16.3	18.3	19.4	20.7	24.3	26.3	29.9
118	20.9563	0.09227	-0.3732	16.5	18.4	19.6	20.9	24.5	26.6	30.3
118.5	21.0925	0.09226	-0.3732	16.6	18.6	19.8	21.1	24.7	26.9	30.6
119	21.3014	0.092223	-0.3732	16.7	18.8	20	21.3	25	27.2	30.9
119.5	21.5963	0.092221	-0.3732	16.8	18.9	20.2	21.6	25.2	27.4	31.2
120	21.8632	0.09215	-0.3732	16.9	19	20.4	21.8	25.4	27.7	31.5

Table (7) shows Egyptian L, M and S parameters and Z score BMI for age for boys from birth to 5 years.

BMI-for-age BOYS<br>Egyptian Z-score Birth to 5 Years											
Y:M	M	Mean	S	L	-3SD	-2SD	-1SD	Median	1SD	2SD	3SD
00:00	0	14.1	0.09853	-0.2587	10.5	11.4	12.5	14.1	15.2	17	19
00:01	1	15.7	0.09741	-0.3025	11.8	12.9	14	15.7	16.8	18.7	20.6
00:02	2	17	0.09623	0.2587	13	14	15.5	17	18.1	20.2	22
00:03	3	17.8	0.09325	0.3698	13.7	14.6	16	17.8	18.9	21	22.6
00:04	4	18.1	0.09111	0.4875	13.9	14.8	16.3	18.1	19.2	21.4	22.9
00:05	5	18.2	0.08952	0.5845	14	14.9	16.4	18.2	19.3	21.5	23
00:06	6	18.2	0.08854	-0.0128	14.1	15	16.5	18.2	19.3	21.5	23
00:07	7	18.2	0.08741	-0.0008	14.1	15.1	16.5	18.2	19.3	21.5	23
00:08	8	18.2	0.08632	-0.1325	14.1	15.1	16.4	18.2	19.2	21.4	22.9
00:09	9	18.2	0.08521	-0.1925	14.1	15.1	16.3	18.2	19	21.3	22.7
00:10	10	18.1	0.08321	-0.2123	14.1	15	16.2	18.1	18.9	21.2	22.5
00:11	11	17.9	0.08264	-0.2963	14	14.9	16.1	17.9	18.8	21.1	22.3
01:00	12	17.8	0.08121	-0.3147	13.9	14.8	16	17.8	18.7	21	22.1
01:01	13	17.8	0.08009	-0.3258	13.8	14.7	15.9	17.8	18.6	20.9	22
01:02	14	17.7	0.07999	-0.3369	13.8	14.6	15.8	17.7	18.5	20.8	22
01:03	15	17.6	0.07951	-0.4123	13.7	14.5	15.7	17.6	18.3	20.7	21.9
01:04	16	17.5	0.07941	-0.4456	13.7	14.4	15.6	17.5	18.2	20.6	21.8
01:05	17	17.4	0.07841	-0.4789	13.6	14.3	15.5	17.4	18.1	20.5	21.8
01:06	18	17.3	0.07831	-0.5258	13.5	14.2	15.4	17.3	18	20.4	21.7
01:07	19	17.3	0.07811	-0.5396	13.5	14.1	15.4	17.3	17.9	20.2	21.6
01:08	20	17.2	0.07799	-0.6014	13.4	14	15.4	17.2	17.8	20	21.5
01:09	21	17.1	0.07788	-0.6023	13.4	14	15.3	17.1	17.7	19.9	21.4
01:10	22	17	0.07655	-0.6123	13.3	13.9	15.3	17	17.6	19.9	21.4
01:11	23	17	0.07511	-0.6258	13.3	13.8	15.2	17	17.5	19.9	21.3
02:00	24	16.9	0.07452	-0.6369	13.3	13.8	15.2	16.9	17.5	19.8	21.3
02:01	25	16.9	0.07489	-0.6321	13.2	13.8	15.2	16.9	17.5	19.8	21.2
02:02	26	16.8	0.07512	-0.6111	13.2	13.8	15.1	16.8	17.5	19.8	21.2
02:03	27	16.8	0.07555	-0.5958	13.2	13.8	15.1	16.8	17.5	19.7	21.1
02:04	28	16.7	0.07599	-0.5825	13.1	13.8	15.1	16.7	17.5	19.7	21.1
02:05	29	16.7	0.07623	-0.5555	13.1	13.7	15	16.7	17.5	19.6	21
02:06	30	16.6	0.07655	-0.5456	13.1	13.7	15	16.6	17.5	19.6	21
02:07	31	16.6	0.07698	-0.4963	13.1	13.7	15	16.6	17.5	19.6	21
02:08	32	16.5	0.07701	-0.4753	13.1	13.7	14.9	16.5	17.4	19.5	20.9
02:09	33	16.5	0.07723	-0.4456	13	13.7	14.9	16.5	17.4	19.5	20.9
02:10	34	16.4	0.07745	-0.4357	13	13.7	14.9	16.4	17.4	19.5	20.8
02:11	35	16.3	0.07799	-0.3258	13	13.7	14.8	16.3	17.4	19.4	20.8
03:00	36	16.2	0.07825	-0.3515	12.9	13.6	14.8	16.2	17.3	19.4	20.8
03:01	37	16.2	0.07855	-0.2963	12.9	13.6	14.8	16.2	17.3	19.4	20.8
03:02	38	16.2	0.07899	-0.2852	12.9	13.6	14.8	16.2	17.3	19.4	20.8
03:03	39	16.1	0.07999	-0.2745	12.8	13.6	14.8	16.1	17.3	19.4	20.7

03:04	40	16.1	0.08006	-0.2632	12.8	13.5	14.8	16.1	17.3	19.4	20.7
03:05	41	16.1	0.08012	-0.2523	12.8	13.5	14.8	16.1	17.3	19.4	20.7
03:06	42	16	0.08036	-0.2456	12.7	13.5	14.7	16	17.3	19.5	20.7
03:07	43	16	0.08054	-0.2321	12.7	13.5	14.7	16	17.2	19.5	20.7
03:08	44	16	0.08111	-0.2258	12.7	13.4	14.7	16	17.2	19.5	20.7
03:09	45	15.9	0.08121	-0.2369	12.6	13.4	14.7	15.9	17.2	19.5	20.7
03:10	46	15.9	0.08151	-0.2456	12.6	13.4	14.7	15.9	17.2	19.5	20.8
03:11	47	15.9	0.08161	-0.2587	12.6	13.4	14.7	15.9	17.2	19.5	20.8
04:00	48	15.8	0.08222	-0.2698	12.5	13.4	14.6	15.8	17.2	19.6	20.8
04:01	49	15.8	0.08299	-0.2758	12.5	13.3	14.6	15.8	17.2	19.6	20.9
04:02	50	15.8	0.08312	-0.2852	12.5	13.3	14.6	15.8	17.2	19.6	20.9
04:03	51	15.8	0.08325	-0.2936	12.5	13.3	14.5	15.8	17.2	19.6	20.9
04:04	52	15.8	0.08365	-0.2999	12.4	13.3	14.5	15.8	17.2	19.6	20.9
04:05	53	15.8	0.08398	-0.3003	12.4	13.3	14.5	15.8	17.1	19.7	20.9
04:06	54	15.7	0.08401	-0.3055	12.4	13.2	14.5	15.7	17.1	19.7	21
04:07	55	15.7	0.08409	-0.3111	12.4	13.2	14.5	15.7	17.1	19.7	21
04:08	56	15.7	0.08412	-0.3199	12.4	13.2	14.4	15.7	17.1	19.7	21.1
04:09	57	15.7	0.08429	-0.3222	12.4	13.2	14.4	15.7	17.1	19.7	21.1
04:10	58	15.7	0.08435	-0.3321	12.4	13.2	14.4	15.7	17.1	19.8	21.2
04:11	59	15.7	0.08488	-0.3411	12.4	13.2	14.4	15.7	17.1	19.8	21.2
05:00	60	15.7	0.08499	-0.3522	12.4	13.2	14.4	15.7	17.1	19.8	21.3

Table (8) shows Egyptian L, M and S parameters and Z score BMI for age for girls from birth to 5 years.

BMI-for-age GIRLS Egyptian Z-score Birth to 5 Years											
Y:M	M	Mean	S	L	-3SD	-2SD	-1SD	Median	1SD	2SD	3SD
00:00	0	14	0.09969	-0.0565	11	11.9	13	14	15	17	18.3
00:01	1	15.2	0.09897	-0.0698	11.6	12.7	14	15.2	16.5	18.2	19.8
00:02	2	16.8	0.09784	-0.0632	12.4	13.6	15	16.8	17.8	19.9	21.5
00:03	3	17.3	0.09625	0.2555	13	14.2	15.8	17.4	18.5	21	22.5
00:04	4	17.7	0.09587	0.3612	13.3	14.5	16	17.7	18.9	21.2	22.7
00:05	5	17.9	0.09422	-0.0198	13.5	14.6	16.2	17.9	19	21.2	22.8
00:06	6	18	0.09325	-0.0874	13.6	14.7	16.3	18	19.1	21.2	22.8
00:07	7	18.1	0.09258	-0.1471	13.6	14.8	16.3	18.1	19.1	21.2	22.8
00:08	8	18.1	0.09125	-0.1589	13.6	14.8	16.2	18.1	19	21.1	22.7
00:09	9	18	0.09088	-0.1987	13.5	14.8	16.1	18	18.9	21	22.6
00:10	10	17.9	0.09008	-0.2589	13.4	14.8	16.1	17.9	18.8	20.9	22.4
00:11	11	17.8	0.08999	-0.3698	13.3	14.7	16	17.8	18.7	20.8	22.3
01:00	12	17.7	0.08987	-0.4789	13.2	14.6	15.9	17.7	18.6	20.7	22.2
01:01	13	17.5	0.08811	-0.5001	13.1	14.5	15.7	17.5	18.4	20.6	22
01:02	14	17.3	0.08741	-0.5017	13	14.4	15.6	17.4	18.3	20.5	21.9
01:03	15	17.3	0.08625	-0.5022	12.9	14.3	15.5	17.3	18.1	20.4	21.8
01:04	16	17.2	0.08502	-0.5111	12.8	14.3	15.4	17.2	17.9	20.4	21.7
01:05	17	17	0.08411	-0.5222	12.7	14.2	15.3	17	17.8	20.3	21.6
01:06	18	16.9	0.08369	-0.5369	12.6	14.1	15.2	16.9	17.8	20.2	21.5
01:07	19	16.9	0.08222	-0.5457	12.6	14	15.1	16.9	17.7	20.1	21.4
01:08	20	16.8	0.08211	-0.5587	12.6	14	15.1	16.8	17.6	20	21.3
01:09	21	16.7	0.08199	-0.5698	12.6	14	15	16.7	17.6	19.9	21.2
01:10	22	16.7	0.08171	-0.5789	12.5	13.9	15	16.7	17.5	19.9	21.1
01:11	23	16.6	0.08152	-0.5871	12.5	13.9	14.9	16.6	17.4	19.9	21
02:00	24	16.6	0.08147	-0.5987	12.5	13.9	14.9	16.6	17.4	19.8	20.9
02:01	25	16.6	0.08125	-0.6001	12.5	13.8	14.9	16.6	17.4	19.8	20.9
02:02	26	16.4	0.08122	-0.6015	12.5	13.8	14.9	16.5	17.4	19.8	20.9
02:03	27	16.5	0.08101	-0.6123	12.5	13.8	14.9	16.5	17.4	19.8	20.9
02:04	28	16.4	0.08099	-0.6128	12.4	13.7	14.9	16.4	17.4	19.8	20.9
02:05	29	16.4	0.08101	-0.6128	12.4	13.7	14.9	16.4	17.4	19.8	20.9
02:06	30	16.3	0.08122	-0.6128	12.4	13.7	14.9	16.3	17.4	19.8	20.8
02:07	31	16.3	0.08166	-0.6128	12.4	13.6	14.9	16.3	17.4	19.7	20.8
02:08	32	16.2	0.08199	-0.6128	12.4	13.6	14.9	16.2	17.4	19.7	20.8
02:09	33	16.2	0.08201	-0.6128	12.4	13.6	14.9	16.2	17.4	19.7	20.8
02:10	34	16.1	0.08222	-0.6128	12.4	13.5	14.9	16.1	17.4	19.7	20.7
02:11	35	16.1	0.08236	-0.6128	12.4	13.5	14.9	16.1	17.4	19.7	20.7
03:00	36	15.9	0.08299	-0.6128	12.4	13.4	14.8	16	17.4	19.7	20.7
03:01	37	16	0.08301	-0.6128	12.4	13.4	14.8	16	17.4	19.7	20.7
03:02	38	16	0.08322	-0.6128	12.4	13.4	14.8	16	17.4	19.7	20.8
03:03	39	16	0.08333	-0.6128	12.3	13.4	14.8	16	17.4	19.7	20.8

03:04	40	15.9	0.08345	-0.6128	12.3	13.4	14.8	15.9	17.4	19.7	20.8
03:05	41	15.9	0.08359	-0.6128	12.3	13.3	14.8	15.9	17.4	19.7	20.9
03:06	42	15.9	0.08366	-0.6128	12.3	13.3	14.7	15.9	17.4	19.7	20.9
03:07	43	15.8	0.08399	-0.6128	12.3	13.3	14.7	15.8	17.4	19.7	20.9
03:08	44	15.7	0.08401	-0.6128	12.2	13.3	14.7	15.8	17.4	19.7	21
03:09	45	15.7	0.08422	-0.6128	12.2	13.2	14.7	15.8	17.4	19.7	21
03:10	46	15.7	0.08435	-0.6128	12.2	13.2	14.7	15.8	17.4	19.7	21.1
03:11	47	15.6	0.08444	-0.6128	12.2	13.2	14.7	15.7	17.4	19.7	21.1
04:00	48	15.7	0.08465	-0.6128	12.2	13.2	14.6	15.7	17.4	19.7	21.2
04:01	49	15.7	0.08475	-0.6128	12.2	13.2	14.6	15.7	17.4	19.8	21.2
04:02	50	15.7	0.08485	-0.6128	12.1	13.2	14.6	15.7	17.4	19.8	21.2
04:03	51	15.7	0.08495	-0.6128	12.1	13.2	14.6	15.7	17.4	19.9	21.3
04:04	52	15.6	0.08499	-0.6128	12.1	13.1	14.6	15.6	17.4	19.9	21.3
04:05	53	15.6	0.08501	-0.6128	12.1	13.1	14.6	15.6	17.4	20	21.4
04:06	54	15.6	0.08511	-0.6128	12.1	13.1	14.5	15.6	17.5	20	21.4
04:07	55	15.6	0.08532	-0.6128	12.1	13.1	14.5	15.6	17.5	20.1	21.4
04:08	56	15.5	0.08555	-0.6128	12.1	13.1	14.5	15.6	17.5	20.2	21.5
04:09	57	15.5	0.08565	-0.6128	12	13.1	14.5	15.5	17.5	20.3	21.5
04:10	58	15.5	0.08578	-0.6128	12	13	14.5	15.5	17.5	20.4	21.6
04:11	59	15.5	0.08625	-0.6128	12	13	14.5	15.5	17.5	20.5	21.6
05:00	60	15.5	0.08789	-0.6128	12	13	14.5	15.5	17.5	20.5	21.7
