# Supplementary material for: Development of LMS and Z Score Growth References for Egyptian Children From Birth Up to 5 Years
Source: Front Pediatr. 2021 Jan 18;8:598499. doi: 10.3389/fped.2020.598499 (PMC7849193; doi:10.3389/fped.2020.598499)
Supplement: Supplementary file 2 [file Data_Sheet_2.PDF]

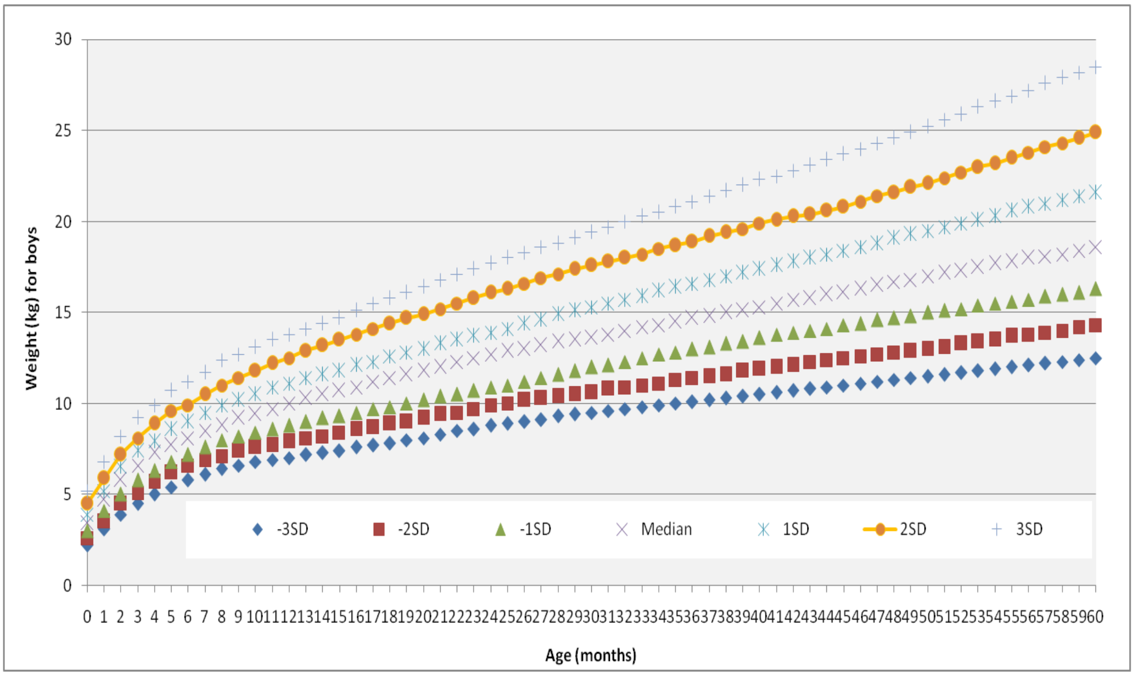

Figure suppl (1): Scatter Plot of Z score weight for age for boys from birth to 5 years

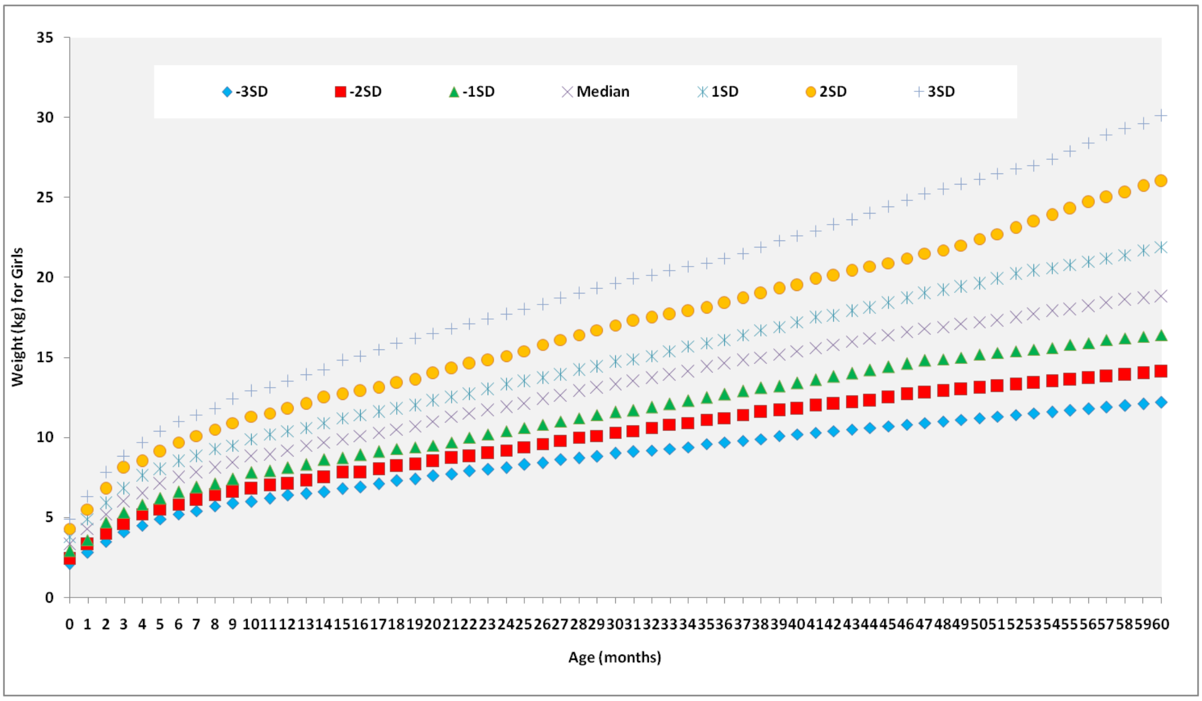

Figure suppl (2): Scatter Plot of Z score weight for age for girls from birth to 5 years

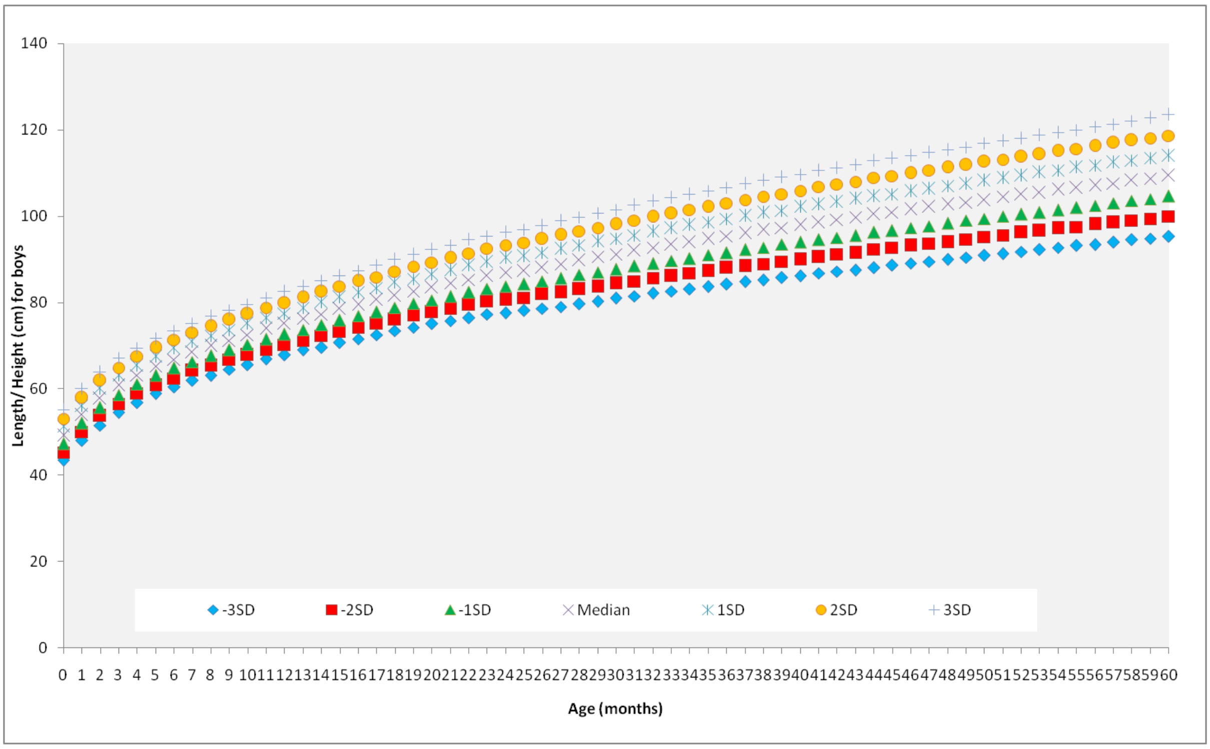

Figure suppl (3): Scatter Plot of Z score length/height for age for boys from birth to 5 years

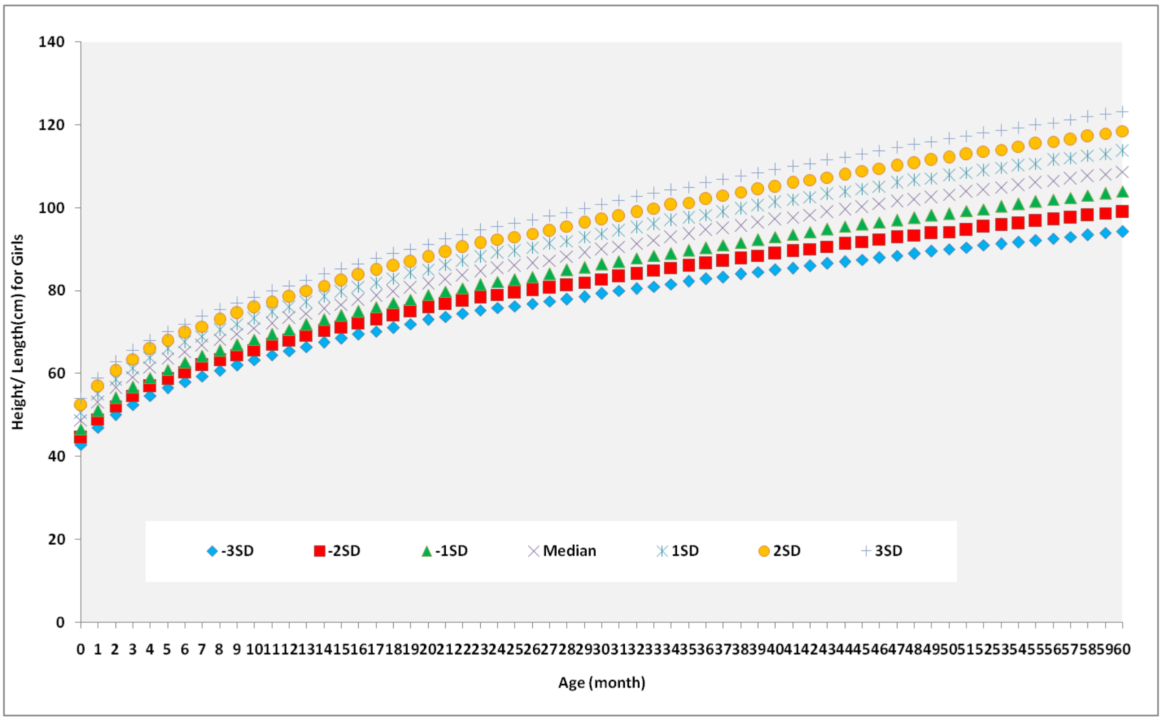

Figure suppl (4): Scatter Plot of Z score length/height for age for Girls from birth to 5 years

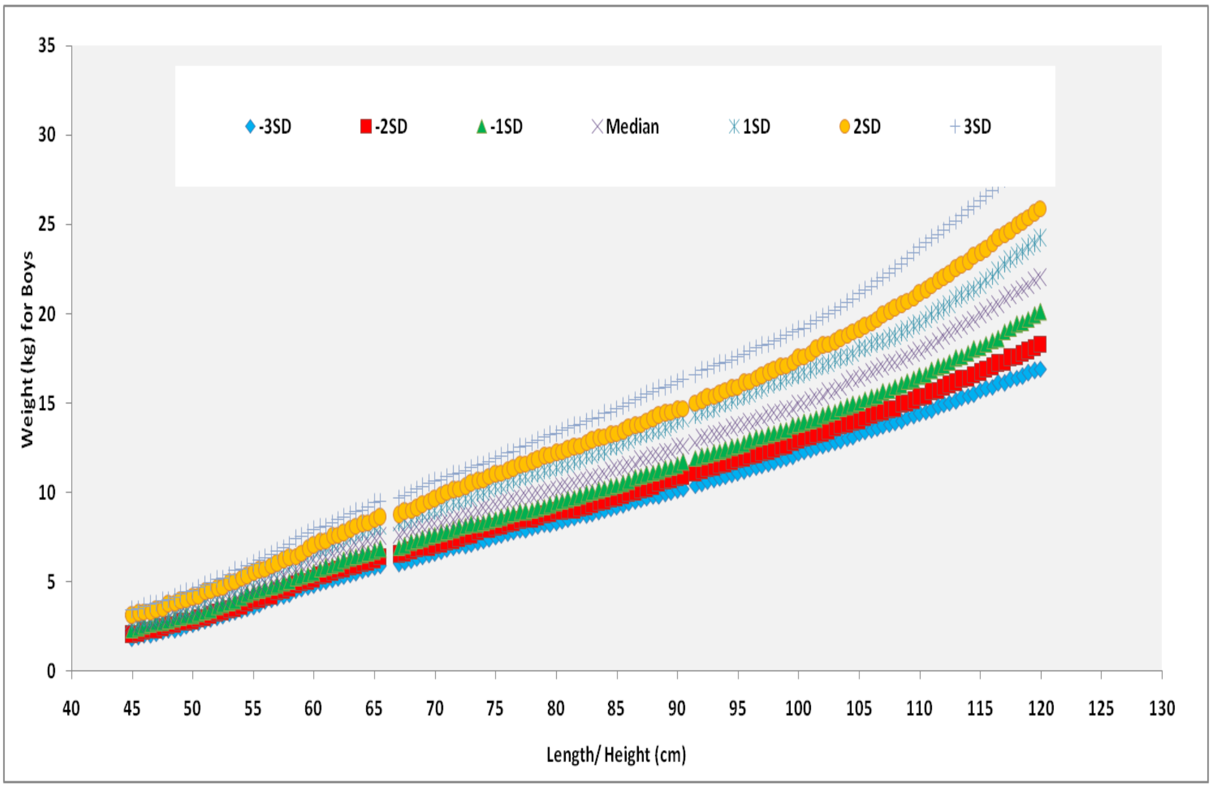

Figure suppl (5): Scatter Plot of Z score Wight for length/height for boys from birth to 5 years

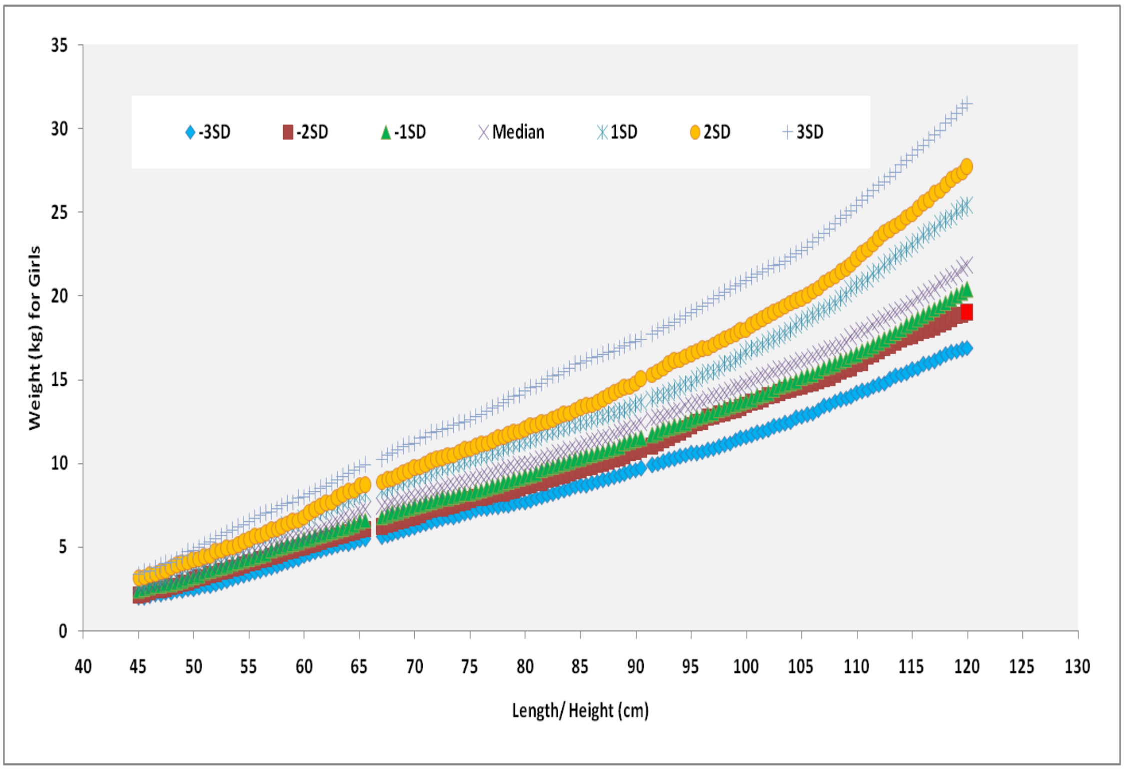

Figure suppl (6): Scatter Plot of Z score Wight for length/height for girls from birth to 5 years

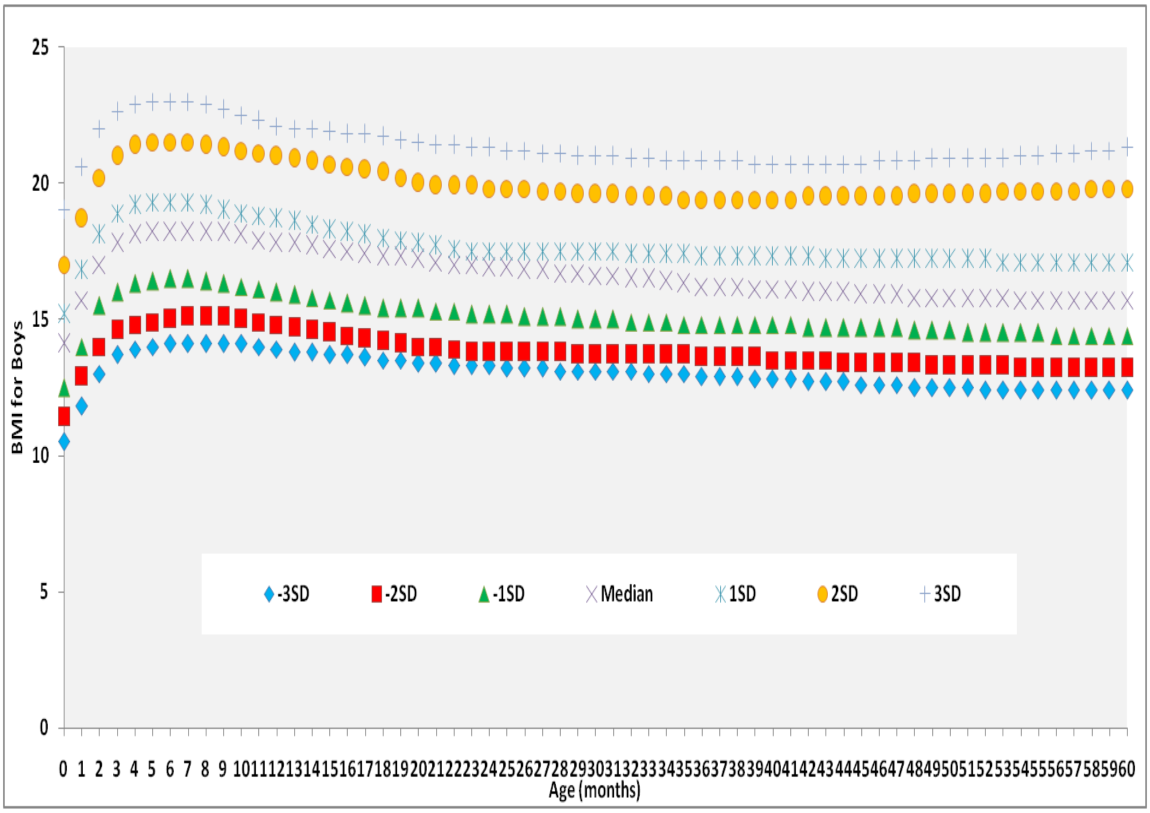

Figure suppl (7): Scatter Plot of Z score BMI for age for Boys from birth to 5 years.

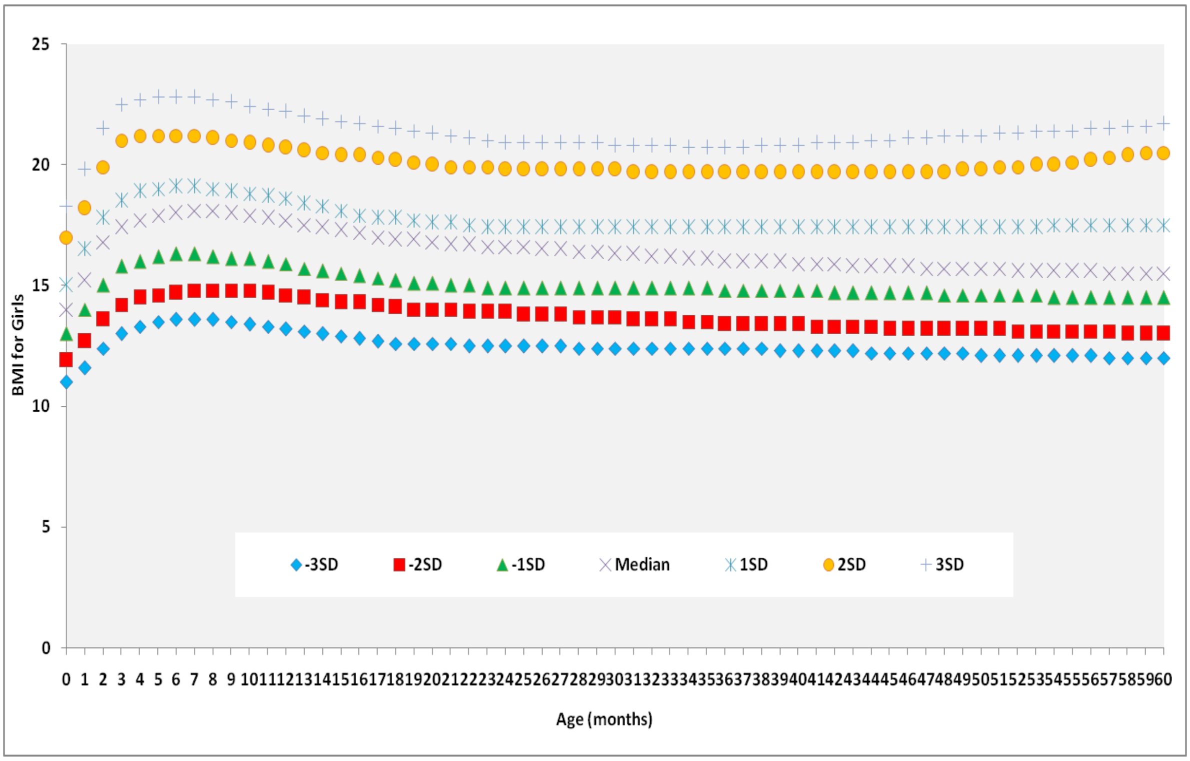

Figure suppl (8): Scatter Plot of Z score BMI for age for Girls from birth to 5 years.
